# Fine-scale haplotype mapping of *MUT*, *AACS*, *SLC6A15* and *PRKCA* genes indicates association with insulin resistance of metabolic syndrome and relationship with branched chain amino acid metabolism or regulation

**DOI:** 10.1371/journal.pone.0214122

**Published:** 2019-03-26

**Authors:** Sara Haydar, Florin Grigorescu, Mădălina Vintilă, Yannick Cogne, Corinne Lautier, Yildiz Tutuncu, Jean Frederic Brun, Jean Marie Robine, Michel Pugeat, Christophe Normand, Patrick Poucheret, Monica Livia Gheorghiu, Carmen Georgescu, Corin Badiu, Nicoleta Băculescu, Eric Renard, Dorina Ylli, Stephanie Badiou, Thibault Sutra, Jean Paul Cristol, Jacques Mercier, Ramon Gomis, Josep Maria Macias, Serghey Litvinov, Elza Khusnutdinova, Catalina Poiana, Renato Pasquali, Davide Lauro, Giorgio Sesti, Sabrina Prudente, Vincenzo Trischitta, Agathocles Tsatsoulis, Sonia Abdelhak, Abdelhamid Barakat, Akila Zenati, Agron Ylli, Ilhan Satman, Timo Kanninen, Yves Rinato, Sasa Missoni

**Affiliations:** 1 University of Montpellier, UMR204 NUTRIPASS (IRD, UM, SupAgro), Montpellier, France; 2 Universitatea de Medicina si Farmacie Carol Davila, Department of Endocrinology, Bucharest, Romania; 3 Istanbul University, Department of Internal Medicine, Istanbul, Turkey; 4 University of Montpellier, PhyMedExp, INSERM, CNRS, Department of Biochemistry and Hormonology, CHRU Montpellier, Montpellier, France; 5 University of Montpellier, INSERM U1198, Montpellier, France; 6 University Claude Bernard de Lyon 1, Lyon-Bron, France; 7 Montpellier University, UMR 95 Qualisud, Montpellier, France; 8 Universitatea de Medicina si Farmacie Iuliu Hatieganu, Cluj-Napoca, Romania; 9 Centre Hospitalier Regional Universitaire de Montpellier, Departement d'Endocrinologie, Diabète, Nutrition, Hôpital Lapeyronie, Montpellier, France; 10 Mjekesise University of Tirana, Tirana, Albania; 11 Institut d'Investigacions Biomediques August Pi i Sunyer, Barcelona, Spain; 12 Institut Catala d'Arqueologia Classica, Tarragona, Spain; 13 Institut biohimii i genetiki RAN, Ufa, Russian Federation; 14 University Alma Mater Studiorum, Division of Endocrinology, Bologna, Italy; 15 Universita degli Studi di Roma Tor Vergata, Roma, Italy; 16 University Magna Graecia of Catanzaro, Catanzaro, Italy; 17 Scientific Institute Casa Sollievo della Sofferenza, San Giovani Rotondo, Italy; 18 University of Ioannina School of Medicine, Department of Endocrinology, Ioannina, Greece; 19 Institut Pasteur de Tunis, Laboratory of Biomedical Genomics and Oncogenetics, Tunis, Tunisia; 20 Institut Pasteur du Maroc, Casablanca, Morocco; 21 Universite d'Alger, CHU Bab-El-Oued, Alger, Algeria; 22 BC Platforms Ltd Oy, Espoo, Finland; 23 Intactile Design SA, Montpellier, France; 24 Institute for Anthropological Research, Zagreb, Croatia; Peking University Third Hospital, CHINA

## Abstract

Branched chain amino acids (BCAA) are essential elements of the human diet, which display increased plasma levels in obesity and regained particular interest as potential biomarkers for development of diabetes. To define determinants of insulin resistance (IR) we investigated 73 genes involved in BCAA metabolism or regulation by fine-scale haplotype mapping in two European populations with metabolic syndrome. French and Romanians (n = 465) were genotyped for SNPs (Affymetrix) and enriched by imputation (BEAGLE 4.1) at 1000 genome project density. Initial association hits detected by sliding window were refined (HAPLOVIEW 3.1 and PHASE 2.1) and correlated to *homeostasis model assessment* (HOMA_IR_) index, in vivo insulin sensitivity (S_I_) and BCAA plasma levels (ANOVA). Four genomic regions were associated with IR located downstream of *MUT*, *AACS*, *SLC6A15* and *PRKCA* genes (P between 9.3 and 3.7 x 10^−5^). Inferred haplotypes up to 13 SNPs length were associated with IR (e.g. *MUT* gene with P < 4.9 x 10^−5^; Bonferroni 1.3 x 10^−3^) and synergistic to HOMA_IR_. SNPs in the same regions were also associated with one order of magnitude lower P values (e.g. rs20167284 in the *MUT* gene with P < 1.27 x 10^−4^) and replicated in Mediterranean samples (n = 832). In French, influential haplotypes (OR > 2.0) were correlated with *in vivo* insulin sensitivity (1/S_I_) except for *SLC6A15* gene. Association of these genes with BCAA levels was variable, but influential haplotypes confirmed implication of *MUT* from *BCAA* metabolism as well as a role of regulatory genes (*AACS* and *PRKCA*) and suggested potential changes in transcriptional activity. These data drive attention towards new regulatory regions involved in IR in relation with BCAA and show the ability of haplotypes in phased DNA to detect signals complimentary to SNPs, which may be useful in designing genetic markers for clinical applications in ethnic populations.

## Introduction

Obesity, type 2 diabetes (T2D) and metabolic syndrome (MetS) are complex disorders characterized by insulin resistance (IR) and severely impact morbidity and mortality of human populations [1, 2]. IR also characterizes several rare genetic syndromes due to inheritable defects in insulin signaling [3]. In complex conditions, however, IR is determined by multiple defects interacting with environmental factors [4]. Branched chain amino acids (BCAA) including leucine (Leu), isoleucine (Ile) and valine (Val) are essential elements of the human diet. Since Felig et al. reported elevated levels in obesity [5], plasma BCAA regained particular interest in relation to IR as predictive biomarkers for the development of diabetes [6–10]. Their elevation in plasma is explained by complex mechanisms since BCAA metabolism is affected by dietary intake, transport and catabolism of BCAA as well as cross-talk between systemic and central nervous system actions [11]. Catabolism of BCAA can be explained by modulation of the rate-limiting step enzyme *branched-chain ketoacid dehydrogenase* (*BCKD*) that decarboxylates derivatives of α-keto acids to acyl-CoA [11]. Recent studies indicate that reduction of BCAA intake in a Western diet was sufficient to improve metabolic health, weight reduction, insulin sensitivity and to transiently induce secretion of the fibroblast growth factor 21 (FGF-21) [12, 13]. Dietary intake of BCAA was also correlated with obesity in Mediterranean populations and with IR or T2D [14–16]. Other emerging data indicated that not only BCAA concentration *per se* but concomitant altered oxidative metabolism, particularly involving the muscle tissue, would be involved in IR [17]. End-products and ketogenic substrates of BCAA catabolism such as β-hydroxybutyrate (β-OHB) have been shown as early biomarkers for IR or glucose tolerance [18]. Moreover, studies in mice and humans recently involved the *metylmalonyl-CoA mutase* (*MUT*) gene (Chr 6p12.3) in BCAA flux abnormalities by modulating fatty acid oxidation in IR individuals [17, 19].

In complex diseases, the existence of genetic defects of BCAA metabolism has been long hypothesized, but the link with IR remained elusive. Mendelian loss-of-function mutations in genes of the BCAA pathway suggest the potential contribution of genetic variants [20]. In humans, the best-known Mendelian mutations concern the *BCKD complex* gene responsible for maple syrup urine disease (MSUD), but many other defects are known (for a review, see Ref [21, 22]. Mendelian mutations in the *MUT* gene, which are responsible for methylmalonic acidemia (MMA) are another examples of severe defects in BCAA metabolism [21]. Some cases of MMA were described associated with severe IR. Although the relation with IR was not further investigated at the molecular level, these rare cases raise new hypothesis of potential role of BCAA metabolism genes and IR [21, 23].

In common forms of obesity and T2D and in absence of coding mutations, the genetics of BCAA metabolism was approached by investigating the correlation between genomic single nucleotide polymorphisms (SNP) and plasma BCAA levels or more specifically, by Mendelian randomization (MR) in Genome Wide Association Studies (GWAS) conformation. Thus, in a large cohort, such MR studies combined with metabolomics revealed the role of the *PPM1K* gene on Chr 4q22.1 that encodes for the phosphatase that activates the *BCKD* complex by dephosphorylating the E1α subunit in mitochondria [24]. Findings were concordant with clinical trials using SNP rs1440581 as a marker, although the effects were found on β-cell secretion rather than IR [25–27]. In another cohort, MR indicated that SNPs for IR as a primary mechanism would be causally related with circulating BCAA levels without excluding however the interference with inflammatory events [28,29]. Therefore, a debate was raised in the scientific literature concerning the sequence of events in inducing changes in BCAA plasma levels and pathogenesis of IR [28].

While these previous studies investigated the genetics of BCAA through the canonical use of independent SNPs in unphased DNA, the European MEDIGENE program (FP7-279171) launched the hypothesis that detection of inheritable defects could benefit from haplotype-mapping on phased DNA (https://cordis.europa.eu/project/rcn/101810_en.html). Haplotypes inferred in phased DNA are combinations of multiple SNPs across the human genome and supposedly better capture the genetic information in diploid individuals at the population level (https://cordis.europa.eu/result/rcn/195753_fr.html).

This study was aimed to perform a fine-scale haplotype mapping of genes of BCAA metabolism or its regulation in two European populations (French and Romanians) affected by MetS with various degree of IR in the frame of MEDIGENE program exploring Mediterranean populations. In a subgroup of French subjects, data were also available to tentatively correlate findings with *in vivo* insulin sensitivity measured by IVGTT and BCAA plasma levels. This paper describes the identification of four genomic regions close to *MUT*, *AACS*, *SLC6A15* and *PRKCA* genes associated with IR and dissects the complex association signal delivered by fine-scale haplotype mapping compared with independent SNPs.

## Patients and methods

### Populations and ethical statement

This study included 465 unrelated subjects with and without MetS from France and Romania (Table 1). Diagnosis of MetS was based on National Cholesterol Education Program (NCEP) and Adult Treatment Panel-III (ATP-III) criteria [30]. National Ethical Committees approved the study protocols, and informed consent was obtained from each patient in accordance with the Helsinki Declaration [31]. Romanian and French collections (MEDIGENE-1) were declared to the Ministère de lʼEnseignement Supérieur de la Recherche et de lʼInnovation (MESRI) under CODECOH # DC-2014-2226 together with Mediterranean collections. Controls were recruited during annual check-ups, and for the French subjects, additional controls were included from the GEHA (Genetics of Health Aging) project [32]. Patient’s data were recorded through a secure portal (https://xz.univ-montp1.fr) using the MEDIPAD software and were stored in Medigene, Anthropological & Genetic DataBase (MAGDB) [14]. For replication, we used an extended sample (n = 832) from Southern Europe (*Turkey*, *Italy*, *Greece and Spain*). IR was assessed using the Homeostasis Model Assessment (HOMA_IR_) index. Insulin resistant (IR) subjects were defined based on fasting insulin + 2 SEM in normal weight controls (cutoff values were 14.6 and 8.9 μU/ml for French and Romanians, respectively). In the subgroup of French subjects, HOMA_IR_ was compared with the insulin sensitivity index (S_I_) from IVGTT and expressed in min^-1^/(μU ml^-1^) x 10^−4^, as previously described [33]. Plasma BCAA were detected by liquid chromatography-mass spectrometry (LC-MS) and expressed in μmol/L.

**Table 1:** Phenotype features of patients with MetS and controls in French and Romanians.

	CTR	MetS	P value^a^
*n*	345	120	NA
Gender (F/M)	312/33	104/16	NS
Age (years)	42.12 ± 1.29	53.15 ± 1.07	< 0.0001
BMI (kg/m^2^)	25.02 ± 0.35	36.06 ± 0.65	< 0.0001
Waist (cm)	89.05 ± 1.37	109.93 ± 1.34	< 0.0001
Fasting Glucose (mmol/L)	4.81 ± 0.9	6.37 ± 1.89	< 0.0001
Fasting insulin (μU/mL)	10.33 ± 1.07	16.52 ±1.13	< 0.0001
Hyperglycemia (%)^b^	10.43	69.16	< 0.0001
HOMA_IR_	2.12 ± 0.21	4.59 ± 0.32	< 0.0001
Insulin resistance (%)^c^	11.04	74.16	< 0.0001
SBP (mmHg)	120.5 ± 2.26	139.55 ± 1.89	< 0.0001
DBP (mmHg)	73.4 ± 1.46	83.72 ± 1.28	< 0.0001
Triglycerides (mmol/L)	1.11 ± 0.69	1.95 ± 0.10	< 0.0001
HDL-cholesterol (mmol/L)	1.39 ± 0.04	1.20 ± 0.03	< 0.0001
Obesity (%)^d^	18.84	81.67	< 0.0001
Hypertension (%)^b^	34.78	90.83	< 0.0001
High Triglycerides (%)^b^	20.29	72.50	< 0.0001
Low HDL (%)^b^	10.14	64.16	< 0.0001

Data are presented as mean ± SEM. Controls and MetS groups were compared using Mann-Whitney test for numerical variable and χ^2^ for nominal variable. Classification by HOMA_IR_ values is indicated in S1 Table.

a, NA stands for non applicable, NS stands for non-significant

b, In defining hyperglycemia, hypertension, high triglycerides and low HDL nominal variable, treatment of pre-diagnosed type 2 diabetes, high blood pressure or dyslipidemia were also considered

c, Insulin resistance was considered as function of HOMA_IR_ values

d, Obesity was considered based on Body Mass Index (BMI) > 30 kg/m^2^

### Genotyping and imputation

Genomic DNA was genotyped with the customized MEDISCOPE chip by Affymetrix (Santa Clara, CA) containing 758,000 SNPs as previously described [34] using facilities of the Biocomputing (BC/SNP_max_) platform (https://bio.tools/BC_SNPmax). Before imputation, all studied genes were at SNP density identical with the regular EUR 500k chip. Genotyping was performed at the IDIBAPS core facilities (Barcelona, Spain) using the GeneTitan instrument. Quality control (QC) was performed following Affymetrix protocols and yielded a mean call rate for SNPs > 97%. SNPs with Minor Allele Frequency (MAF) < 0.01 or divergent from Hardy-Weinberg (HW) equilibrium (P < 10^−4^) were excluded. Principal Component Analysis (PCA) was performed on 654,000 SNPs, merged with HapMap data and pruned (r^2^ > 0.8). For imputation, genotyped data were imputed with BEAGLE 4.1 program using the 1000 Genomes Project (Phase III) (www.1000genomes.org/data) as SNP reference panel [35]. Imputed SNPs (conform-gt) were filtered for accuracy metrics (AR^2^ > 0.86) and MAF > 0.01 [36, 37]. Genomic locations (GRCh37/hg19, by February 2009) were retrieved from the Axiom Analysis Suite 1.1 from the Mapfile (MEDISCOPE) and then extended to 10 kb upstream and downstream.

### Gene panel of BCAA

To investigate genes in the BCAA metabolism, the main bulk of genes (n = 48) were selected from the KEGG database for BCAA metabolism (http://www.genome.jp/kegg/) to which we added 8 genes for BCAA transport and another 23 for regulation. Some genes from KEGG database (e.g. *AACS)* although not directly involved in BCAA catabolism were included. Other genes for regulation were included based on search in NCBI using query terms *branched chain amino acids* or BCAA in *Homo Sapiens* (human) together with: metabolism, synthesis, degradation, regulation, modification, mitochondrial, metabolic syndrome, obesity, insulin, activity and transport. Imputation failed for 4 genes (*ACSF3*, *HADHA*, *HMGCS1* and *DDX19A*) because of insufficient SNP number in the initial data set. Similarly, 6 genes were absent from our MapFile and therefore excluded from further studies. Thus, we end up with 73 candidate genes to be studied, which contained in total 29,087 SNPs (S1 Table).

### Haplotype-mapping

Haplotype mapping was conducted in two steps. In the first step, to identify the most significant hits for association, we applied a sliding window procedure using SNP & Variation Suite v8.4.4 program (Golden Helix. Inc., Bozeman, MT, www.goldenhelix.com) with a length of 4 SNPs, the expectation/maximization (EM) algorithm for haplotype inference (frequency threshold of 0.01) and Gabriel’s method for linkage disequilibrium (LD) blocks [38]. In a second step, after location of positive hits among genes, longer haplotypes of multiple SNPs were analyzed in HAPLOVIEW 3.1 [39]. In this program, LD blocking was checked and refined and the association signal was analyzed in detail. When indicated we also used the “four gamete” rule for LD blocking. As a rule, we extended analysis of the initial hit with one LD block upstream and downstream in the respective gene. Haplotype pairs were assigned to individuals in the population by PHASE 2.1 program [40]. After the location of regions of interest, a number of 104 SNPs corresponding to these regions were used to compare the haplotype approach with that of independent SNPs in association, replication and power calculation. The power calculated (PBAT) in the same Golden Helix program, which indicated that a power at 0.85 would be expected from SNPs with MAF > 0.2 and displaying effects with OR ≥ 2.5.

### Statistical and computational methods

Associations were tested using logistic regression, including basic, additive and recessive models in SNP & Variation Suite v8.4.4 program. To correct for population stratification, we used the “genomic control” method by calculating the *inflation factor* (λ) among Southern European populations. Significance was established when P was less than 0.05 after *Bonferroni* and *False Discovery Rate* (FDR) corrections. Haplotypes were tested in permutation test using Golden Helix as well as in HAPLOVIEW after 10k reshuffling. Numerical variables (HOMA_IR_, 1/S_I_, BMI, glycaemia, TG, HDL and BCAA levels) were tested using the correlation trend test (Golden Helix) or in ANOVA with a 2α level set at 5% (Statview 5.0, SAS, Abacus Concepts, Berkeley, CA), as described [41–43]. In ANOVA, statistical significance was determined at *2n* chromosomes and for plasma BCAA level, extreme values ± 2SD for the entire population were excluded, while significance among groups was determined by *a posteriori Student-Newman-Keuls* test. To correct HOMA_IR_ values for the body mass index (BMI), we calculated predicted values of the HOMA_IR_ as a function of BMI in each ethnic sample and separately in men and women and then residuals were calculated as previously shown [41]. Values of S_I_ were obtained using the minimal model calculation from IVGTT and tested as 1/S_I_ as previously reported [33]. HaploReg v.4.1 program was used to predict transcriptional activity of SNPs in each haplotype (http://archive.broadinstitute.org/mammals/haploreg).

## Results

### Phenotype

**Fig 1:**
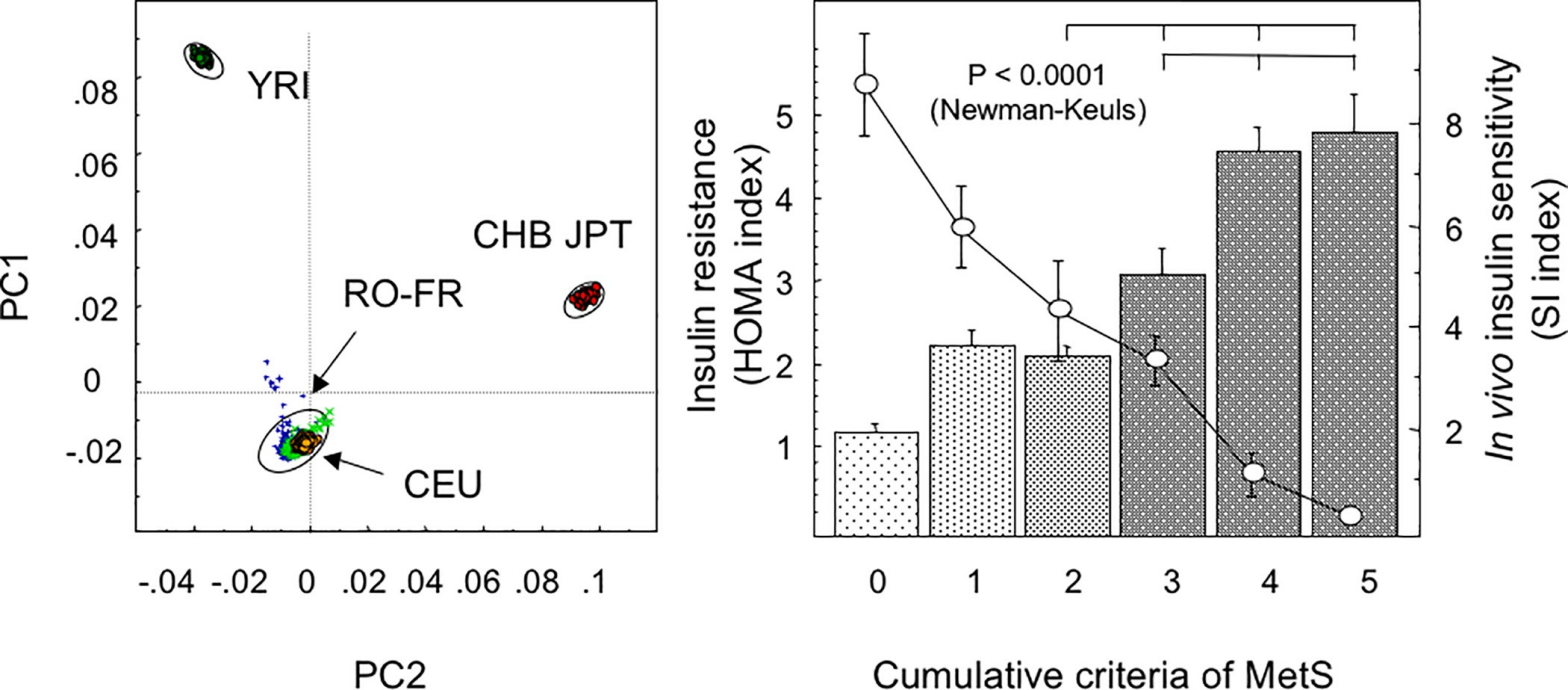
Demographic and clinical characteristics of French and Romanians with MetS. *Left panel*, genetic distances between French and Romanian samples and HapMap populations. PCA was performed on 654,000 SNPs, merged with HapMap data and pruned (R^2^ > 0.8) and eigenvectors were plotted as PC1/PC2. The 95% tolerance ellipse was constructed using the JMP program. YRI, CHB, JPT and CEU stand for Yoruba in Ibadan, Nigeria, Han Chinese in Beijing, Japanese in Tokyo, Utah residents with Northern and Western European ancestry from the CEPH collection, respectively. *Right panel*, relationship between cumulative criteria for MetS and HOMA_IR_ index in French and Romanian subjects. The curve represents the variation of the sensitivity index (S_I_) obtained in a French population using the minimal model calculation after in vivo IVGTT.

Features of patients with and without MetS after exclusion of outliers (n = 12) from PCA are indicated in Table 1 and Fig 1 (left panel). There was a good correlation between the cumulative criteria of MetS and progressive increased HOMA_IR_ index with significant differences between the group with 2 criteria and the group with 3 or more criteria, which define the MetS (Fig 1, right panel). In the French sub-population, this correlation was confirmed by *in vivo* variation of the S_I_ during IVGTT (Spearman correlation with HOMA_IR_, P < 0.0001). No significant differences were detected between French and Romanians for the prevalence of components of MetS. However, the French subjects were more obese (P < 0.0001 in χ^2^ for obesity), while the Romanians had higher values of HOMA_IR_ (3.46 ± 0.25 versus 2.74 ± 0.29, respectively). Since IR *per se* is not included in the ATP^III^ criteria for MetS, this genetic study was conducted by comparing insulin resistant (IR) subjects and non-insulin resistant (non-IR) controls with clinical and biological profiles as indicated in S2 Table. As expected, subjects with IR were more obese: they had higher waist circumferences, and their HOMA_IR_ values were 3-fold higher than non-IR subjects.

### Genetic study

To define genes suitable for haplotype-mapping, we selected a panel of 73 genes from the BCAA pathway and immediate regulators (S1 Table). To detect the most associated genes with IR, we applied the sliding window procedure with a window length of 4 SNPs after identifying LD blocks by Gabriel et al. method [38]. Thus, a number of 109,754 short haplotypes were tested against IR and significant associations (P ≤ 9.39 x 10^−5^) were obtained for 21 haplotypes in 6 genomic regions of Chr 6, 12, 16 and 17 (S3 Table). Among genes of BCAA metabolism defined in the KEGG database, associations were found with downstream genomic regions of the *MUT* and *AACS*. All other genes in BCAA metabolism including *BCAT1* and *BCAT2*, *BCKDHA*, *DBT*, *DLD* displayed hits with at least one order of magnitude lower P values and therefore were not further investigated in fine-scale haplotype mapping. Among genes investigated for their implication in transport of BCAA or its regulation, positive associations were found for *SLC6A15* and *PRKCA* genes. The *PPM1K*, previously implicated by MR studies [24] was not significantly associated with IR and excluded from further analysis. Excluded were also genes for which initial hits (4 SNPs haplotype length) were found only in controls (*FAM46A* and *BCKDHB*) (S3 Table) or for which corresponding SNPs were not sustained by the Bonferroni correction and/or FDR. For instance, *ABAT* gene was excluded because the strongest association displayed by rs56238963 on Chr 16 was with P-value of 0.012, not accepted by Bonferroni and with FDR correction of 0.0518. Another signal at the level of *IGF-1* on Chr 12 was also excluded because there was no association with IR but only with MetS displaying a P-value < 7.80 x 10^−5^ (S3 Table). Finally, we focused for fine-scale haplotype mapping on four genomic regions of the *MUT* gene involved in distal steps of BCAA catabolism, *AACS* involved in ketone bodies metabolism, *SLC6A15* involved in BCAA transport in the brain and its immediate regulator, the *PRKCA* gene. These genomic regions contained in total 104 independent SNPs (S4 and S5 Tables).

### Fine-scale haplotype mapping

**Fig 2:**
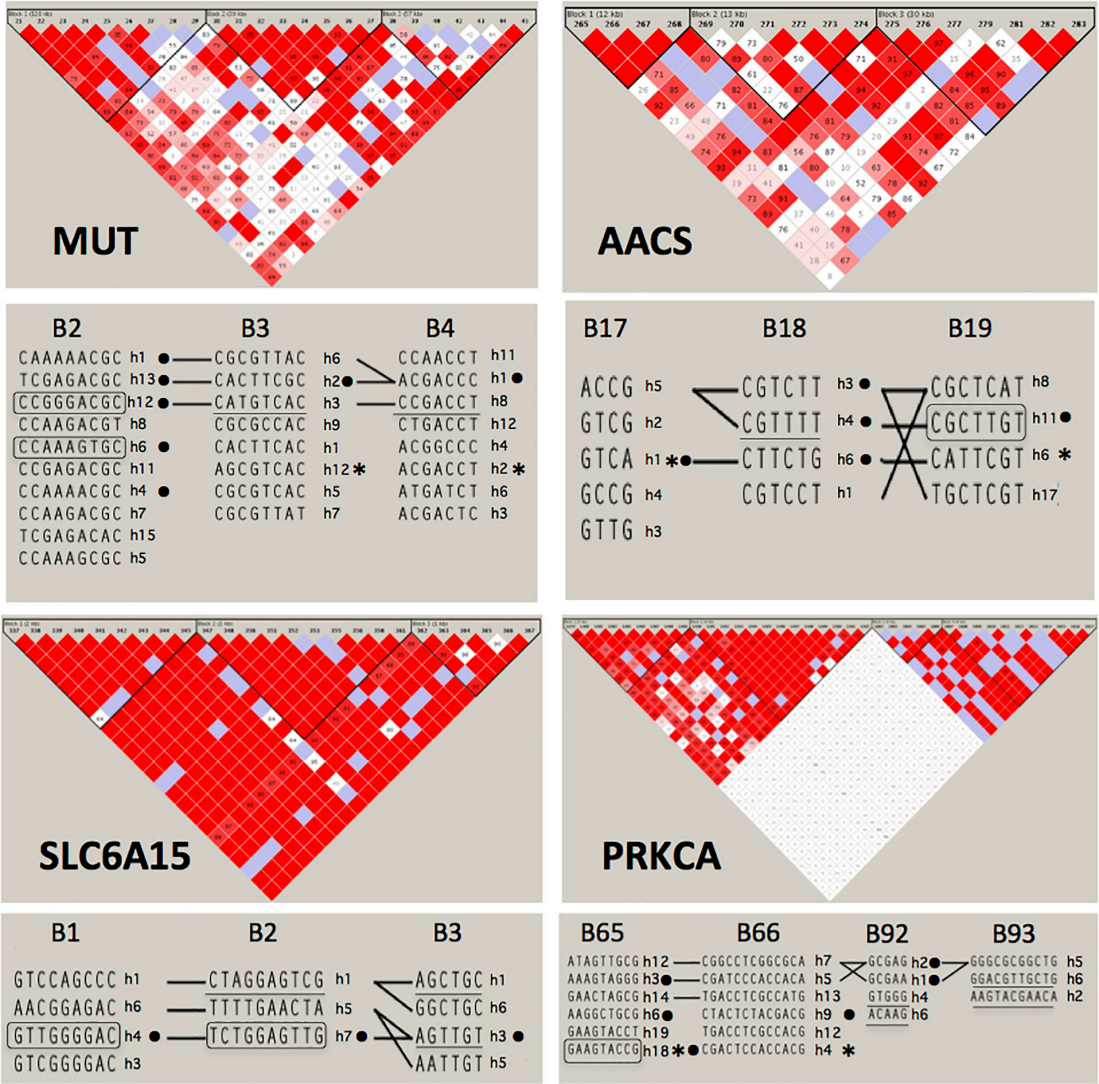
*Linkage disequilibrium* LD blocks and haplotypes in four genes of BCAA metabolism in French and Romanians. Files (.ped and .map) were transferred in the HAPLOVIEW program, and LD blocking was performed with the Gabriel et al. method [38] and/or « four gamete rule ». HW cut off value was fixed at 1 x 10^−4^ and lines inter-blocks were fixed at 0.01. Underlined haplotypes are those significantly associated with HOMA_IR_ values. “*” indicates association with in vivo IR during IVGTT; “•” indicates haplotypes effective on BCAA plasma levels. Haplotypes concordantly associated with IR and BCAA levels are indicated by box. All frequent and rare haplotypes are provided in S6 Table.

Because the sliding window procedure identified positive hits composed of short haplotypes of only 4 SNPs, to define longer haplotypes within LD blocks in population haplotype-mapping was refined in HAPLOVIEW 3.1 and PHASE 2.1. Fine-scale mapping was conducted on Chr 6 (target region 48,279,728–48,292,580) downstream of *MUT* and upstream of *C6orf138* genes. On Chr 12 (84,569,856–84,572,498) we studied the region downstream of *SLC6A15* and the region between positions 125,642,567–125,661,436 located downstream of *AACS* and upstream of *TMEM132B* gene. On Chr 17, the region between 64,783,930–64,795,285 was intra-genic and downstream of PRKCA gene. After inspection of the Manhattan plots, the corresponding genomic regions that contained the association signal were visualized in HAPLOVIEW (after transfer of .ped files from Golden Helix program) and submitted to detailed analysis. As a rule, we studied the LD block containing the leader hit (obtained in the sliding window) and one block upstream and downstream. In HAPLOVIEW, for *PRKCA* two additional LD-blocks were added after signal analysis in HAPLOVIEW. Visualized haplotypes in HAPLOVIEW were also obtained by PHASE 2.1 program. Their sequences are indicated in S6 Table, while LD blocking structure with the most frequent haplotypes (> 0.01) is depicted in Fig 2. The most significant haplotypes associated with IR, including those sustained by the Bonferroni correction and positively correlated in ANOVA with HOMA_IR_ index values are indicated in Table 2.

**Table 2:** Significant associated haplotypes with IR in four genomic regions in French and Romanians.

Haplotype ID (n)^a^	Sequence	Frequency	OR	95% CI		P-value Regression	Bonferroni	FDR	Permutation	HOMA_IR_		P-value ANOVA
				Lower	Upper					non-carriers^b^	carriers	
***MUT***												
B2_H12 (120)	CCG**G**GACGC	0.12	0.47	0.27	0.81	2.82 x 10^−3^	NS	2.45 x 10^−2^	NS	2.94 ± 0.11	2.50 ± 0.25	NS
**B3_H3** (196)	CA**T**GTCAC	0.21	0.45	0.30	0.68	**4.90 x 10**^**−5**^	**1.30 x 10**^**−3**^	1.30 x 10^−3^	4.00 x 10^−3^	**2.99 ± 0.12**	**2.41 ± 0.20**	**3.57 x 10**^**−2**^
B4_H1 (330)	ACGACCC	0.36	1.37	1.01	1.85	4.42 x 10^−2^	NS	NS	NS	2.90 ± 0.13	2.84 ± 0.16	NS
**B4_H8** (201)	**C**CGACC**T**	0.22	0.51	0.34	0.76	**6.44 x 10**^**−4**^	**5.15 x 10**^**−3**^	5.15 x 10^−3^	7.00 x 10^−3^	**3.01 ± 0.12**	**2.40 ± 0.18**	**2.17 x 10**^**−2**^
***AACS***												
B17_H3 (53)	GT**T**G	0.05	0.26	0.10	0.67	8.57 x 10^−4^	1.54 x 10^−2^	5.10 x 10^−3^	1.60 x 10^−2^	2.91 ± 0.10	2.25 ± 0.49	NS
B18_H3 (320)	CGTCTT	0.35	0.56	0.41	0.78	2.92 x 10^−4^	5.30 x 10^−3^	2.60 x 10^−3^	7.00 x 10^−3^	2.98 ± 0.12	2.68 ± 0.19	NS
**B18_H4** (251)	CGT**T**TT	0.27	1.89	1.38	2.59	**1.24 x 10**^**−4**^	**2.20 x 10**^**−3**^	2.20 x 10^−3^	2.00 x 10^−3^	2.71 ± 0.11	3.30 ± 0.22	**1.13 x 10**^**−2**^
B19_H8 (319)	CGCTC**A**T	0.34	0.65	0.47	0.90	8.23 x 10^−3^	4.94 x 10^−2^	1.65 x 10^−2^	NS	2.99 ± 0.13	2.67 ± 0.17	NS
B19_H9 (14)	CGCTCGT	0.01	3.63	1.16	11.37	3.49 x 10^−2^	NS	NS	NS	2.87 ± 0.10	3.53 ± 0.56	NS
B19_H11 (238)	CGCT**T**GT	0.25	1.57	1.14	2.18	6.38 x 10^−3^	3.83 x 10^−2^	1.91 x 10^−2^	4.00 x 10^−2^	2.80 ± 0.12	3.10 ± 0.21	NS
B19_H17 (110)	**T**GCTCGT	0.11	0.42	0.24	0.73	1.33 x 10^−3^	8.01 x 10^−3^	8.01 x 10^−3^	1.50 x 10^−2^	2.89 ± 0.11	2.81 ± 0.40	NS
***SLC6A15***												
B1_H1 (447)	GTCCAGCCC	0.48	1.52	1.13	2.03	3.71 x 10^−3^	NS	3.15 x 10^−2^	NS	2.70 ± 0.14	3.07 ± 0.15	NS
B1_H4 (153)	GT**TGG**G**GA**C	0.16	0.60	0.39	0.92	1.79 x 10^−2^	NS	NS	NS	2.96 ± 0.11	2.48 ± 0.23	NS
**B2_H1** (403)	CTAGGAGTCG	0.44	1.74	1.30	2.33	**2.97 x 10**^**−4**^	**5.10 x 10**^**−3**^	5.10 x 10^−3^	5.00 x 10^−3^	**2.65 ± 0.13**	**3.17 ± 0.16**	**1.56 x 10**^**−2**^
B2_H7 (153)	**TCT**GGAGT**T**G	0.16	0.60	0.40	0.93	1.79 x 10^−2^	NS	4.78 x 10–2	NS	2.96 ± 0.11	2.48 ± 0.23	NS
**B3_H1** (271)	AGCTGC	0.29	1.55	1.13	2.12	**7.10 x 10**^**−3**^	NS	3.02 x 10^−2^	NS	**2.73 ± 0.12**	**3.23 ± 0.20**	**3.26 x 10**^**−2**^
**B3_H3** (259)	AG**T**TG**T**	0.27	0.61	0.43	0.87	**4.63 x 10**^**−3**^	NS	2.63 x 10^−2^	NS	**3.02 ± 0.12**	**2.50 ± 0.18**	**3.41 x 10**^**−2**^
***PRKCA***												
B65_H18 (43)	**G**AAGTA**C**CG	0.04	2.44	1.31	4.52	6.19 x 10^−3^	NS	4.54 x 10^−2^	NS	2.85 ± 0.10	3.48 ± 0.51	NS
B66_H4 (67)	CGACTCCACCAC**G**	0.07	1.82	1.08	3.04	2.42 x 10^−2^	NS	NS	NS	2.87 ± 0.11	2.99 ±	NS
B66_H7 (364)	CG**G**CCTCG**G**C**G**CA	0.39	0.68	0.50	0.93	1.10 x 10^−2^	NS	NS	NS	2.91 ± 0.13	2.83 ±	NS
B66_H12 (69)	TGACCTCGCCAC**G**	0.07	1.70	1.02	2.83	4.81 x 10^−2^	NS	NS	NS	2.87 ± 0.11	2.98 ± 0.37	NS
B92_H1 (388)	GCGA**A**	0.41	1.36	1.01	1.81	3.40 x 10^−2^	NS	NS	NS	2.74 ± 0.13	3.09 ± 0.17	NS
B92_H4 (62)	G**T**G**G**G	0.06	1.83	1.08	3.10	2.42 x 10^−2^	NS	NS	NS	2.81 ± 0.10	3.85 ± 0.51	1.12 x 10^−2^
**B92_H6** (53)	**A**C**A**AG	0.05	0.20	0.07	0.56	**1.93 x 10**^**−4**^	**4.20 x 10**^**−3**^	4.20 x 10^−3^	5.00 x 10^−3^	**2.94 ± 0.11**	**1.79 ± 0.19**	**1.84 x 10**^**−2**^
B93_H2 (61)	**AA**G**TA**CG**AACA**	0.06	1.73	1.02	2.94	4.64 x 10^−2^	NS	NS	NS	2.81 ± 0.10	3.88 ± 0.52	9.30 x 10^−3^
**B93_H6** (46)	GG**A**CG**TT**GCTG	0.05	0.22	0.08	0.62	**6.23 x 10**^**−4**^	**1.37 x 10**^**−2**^	6.90 x 10^−3^	1.60 x 10^−2^	**2.93 ± 0.11**	**1.84 ± 0.22**	**3.95 x 10**^**−2**^

Haplotypes were reconstructed by PHASE 2.1 program, visualized in HAPLOVIEW and tested for association in logistic regression in SNP &Variation Suite v8.4.4. Association with HOMA_IR_ values in carriers and non-carriers was performed by ANOVA. Independent SNPs associated with IR were mapped on each haplotype. Only haplotypes with at least one significant P-value (Bonferroni or FDR) are indicated while all inferred haplotypes, including rare haplotypes (< 0.01) are indicated in S6 Table. Haplotypes sustained by Bonferroni correction or correlated in ANOVA with HOMA_IR_ are indicated in bold.

^a^n is expressed at 2n chromosomes

^b^ Values are expressed as mean ± SEM; Independent SNPs with positive association values are indicated in corresponding haplotype (underlined and bold) and were: *MUT* B2_H12 for rs17674678 (G/A), B3_H3 for rs2167284 (T/C), B4_H8 for rs325041 (C/A) and rs325286 (T/C), *AACS* B17_H3 for rs73233312 (T/C), B18_H4 for rs4442602 (T/C), B19_H8 for rs61943077 (A/G), B19_H11 for rs12818316 (T/C), B19_H17 for rs10846850 (T/C), *SLC6A15* B1_H4 for rs732438 (T/C), rs2403183 (G/C), rs2403184 (G/A), rs1384320 (G/C), rs1384321 (A/C), B2_H7 for rs1482441 (T/C), rs7301137 (C/T), rs10862831 (T/A), rs10779081 (T/C), B3_H3 for rs10779083 (T/C), rs1482429 (T/C), *PRKCA* B65_H18 for rs17762314 (G/A), rs9902356 (C/G), B66_H4 for rs7220480 (G/A), B66_H7 for rs7224351 (G/A), rs2286958 (G/C), rs8072920 (G/A), B66_H12 for rs7220480 (G/A), B92_H1 for rs12603061 (A/G), B92_H4 for rs7208993 (T/C), rs71379997 (G/A), B92_H6 for rs78518692 (A/G) and rs4791033 (A/G), B93_H2 for rs9910304 (A/G), rs35200121 (A/G), rs36011047 (T/C), rs9898120 (A/G), rs16960252 (A/G), rs34169044 (A/C), rs8077180 (C/T), rs9892428 (A/G), B93_H6 for rs118009757 (A/G), rs28450079 (T/C) and rs2362711 (T/G)

Among gene candidates of BCAA metabolism, in the *MUT* gene, there were 4 haplotypes associated with IR, but only B3_H3 and B4_H8 were supported by Bonferroni correction and had concordant associations with HOMA_IR_. Thus, the dominant effect on IR defined by HOMA_IR_ values was found for LD blocks B3 and B4 (Fig 2, left-upper panel, underlined haplotypes). The SNP rs2167284 (T/C) the most significantly associated with IR (T allele) was located on the unique haplotype B3_H3. Influential haplotypes had OR of 0.45 and 0.51, respectively and with moderate decrease in HOMA_IR_ values (19.4 and 20.3%). Another haplotype B4_H1 with OR 1.37 was associated with IR but non statistically significant on HOMA_IR_ values. To confirm the association with IR by *in vivo* measurement, we calculated haplotypes in French population where data on S_I_ values were available. There were no major differences in haplotype prevalence among French and Romanian populations. For M*UT* gene, differences were found for B2_H12 (28.89% versus 15.92%, P < 0.002 in χ^2^ respectively) and for B3_H3 (41.23% versus 29.93%, P < 0.0197, respectively). As shown in Table 3, the two protective haplotypes B3_H3 and B4_H8 were confirmed by variation of 1/S_I_ values *in vivo*, but not significant, while statistical significance was obtained for two pathogenic rare haplotypes B3_H12 and B4_H2 (frequency 0.08 and 0.07) in blocks B3 and B4 with major effect (OR of 2.8 and 3.0). These data indicated that *MUT* gene was associated with IR measured by both HOMA_IR_ or S_I_ values *in vivo*, the major signal for IR being contained in blocks B3 and B4.

For the *AACS* gene, the signal was more complex (Fig 2, right-upper panel). There were 7 associated haplotypes, 6 supported by Bonferroni and 5 containing significant SNPs (Table 2). However, only B18_H4 displayed a significantly increased HOMA_IR_ (underlined in Fig 2). The homozygous carriers (H4/H4 pair) were associated with IR (P < 0.0021) with OR of 2.84, 95% CI [1.45–5.53]. In French population, B17_H1 and B19_H6 with strong effect on association (OR > 2) were confirmed by significant variation of 1/S_I_ (Table 3).

**Table 3:** Significant haplotypes associated with IR in French population and correlation with *in vivo* insulin sensitivity.

Haplotype ID (n)^a^	Frequency	OR	95% CI		P-value Regression^b^	Bonferroni correction	HOMA-IR		1/ S_I_		P-value^d^ ANOVA
			Lower	Upper			non-carriers^c^	carriers^c^	non-carriers^c^	carriers^c^	
***MUT***											
B2_H6 (53)•	0.08	0.13	0.02	0.99	5.91 x 10^−3^	NS	2.57 ± 0.14	1.80 ± 0.15	0.86 ± 0.15	0.30 ± 0.06	3.38 x 10^−1^
B3_H3 (146)	0.24	0.34	0.16	0.73	1.22 x 10^−3^	3.16 x 10^−2^	**2.71 ± 0.17**	**1.88 ± 0.14**^**$**^	0.85 ± 0.16	0.75 ± 0.27	7.73 x 10^−1^
B3_H12 (51)•	0.08	2.82	1.42	5.59	5.63 x 10^−3^	NS	2.49 ± 0.14	2.90 ± 0.52	**0.75 ± 0.14**	**1.52 ± 0.64**	1.53 x 10^−2^*
B4_H2 (46)•	0.07	3.07	1.53	6.17	3.00 x 10^−3^	2.40 x 10^−2^	2.46 ± 0.14	3.25 ± 0.56	**0.74 ± 0.13**	**1.59 ± 0.67**	1.46 x 10^−2^*
B4_H8 (146)	0.24	0.33	0.15	0.70	1.60 x 10^−3^	1.28 x 10^−2^	**2.74 ± 0.17**	**1.84 ± 0.13**^**$**^	0.87 ± 0.17	0.69 ± 0.25	5.83 x 10^−1^
***AACS***											
B17_H1 (177)•	0.28	2.25	1.38	3.67	1.48 x 10^−3^	2.52 x 10^−2^	2.24 ± 0.16	2.71 ± 0.26	**0.62 ± 0.13**	**1.17 ± 0.30**	9.30 x 10^−3^*
B17_H3 (39)	0.06	0.18	0.02	1.30	2.29 x 10^−2^	NS	2.52 ± 0.13	2.66 ± 1.03	0.84 ± 0.14	0.50 ± 0.13	5.96 x 10^−1^
B18_H6 (94)•	0.14	2.01	1.10	3.66	3.12 x 10^−2^	NS	2.47 ± 0.15	2.77 ± 0.35	0.78 ± 0.16	0.97 ± 0.30	6.04 x 10^−1^
B19_H6 (97)•	0.15	2.42	1.38	4.26	3.23 x 10^−3^	1.61 x 10^−2^	2.49 ± 0.15	2.70 ± 0.33	**0.88 ± 0.17**	**0.59 ± 0.15**	3.54 x 10^−2^*
B19_H17 (76)	0.12	0.36	0.13	1.05	2.50 x 10^−2^	NS	2.52 ± 0.14	2.57 ± 0.66	0.85 ± 0.15	0.46 ± 0.15	4.68 x 10^−1^
***SLC6A15***											
B1_H1 (286)	0.46	1.66	1.02	2.70	3.08 x 10^−2^	NS	2.42 ± 0.18	2.66 ± 0.21	0.65 ± 0.15	1.01 ± 0.24	2.03 x10^-1^
B2_H1 (256)	0.41	1.73	1.07	2.81	2.25 x 10^−2^	NS	2.36 ± 0.17	2.77 ± 0.24	0.63 ± 0.14	1.11 ± 0.27	9.45 x 10^−2^
B3_H5 (70)•	0.11	0.41	0.14	1.15	4.24 x 10^−2^	NS	2.52 ± 0.14	2.58 ± 0.51	0.85 ± 0.15	0.62 ± 0.26	6.17 x 10^−1^
***PRKCA***											
B65_H18 (28)	0.04	4.43	1.96	10.0	1.19 x 10^−3^	2.00 x 10^−2^	2.46 ± 0.14	3.44 ± 0.70	**0.74 ± 0.13**	**1.79 ± 0.99**	1.00 x 10^−4^*
B66_H4 (43)	0.06	2.76	1.32	5.76	1.00 x 10^−2^	NS	2.48 ± 0.14	3.05 ± 0.57	**0.70 ± 0.12**	**1.97 ± 0.88**	1.00 x 10^−4^*
B92_H1 (258)	0.41	1.68	1.04	2.74	4.24 x 10^−2^	NS	2.38 ± 0.16	2.76 ± 0.25	0.76 ± 0.18	0.92 ± 0.21	5.97 x 10^−1^
B92_H6 (41)	0.06	0.16	0.02	1.21	1.69 x 10^−2^	NS	2.58 ± 0.14	1.73 ± 0.16	0.86 ± 0.15	0.34 ± 0.06	3.27 x 10^−1^
B93_H6 (36)	0.06	0.18	0.02	1.29	2.52 x 10^−2^	NS	2.57 ± 0.14	1.77 ± 0.18	0.85 ± 0.15	0.34 ± 0.07	3.77 x 10^−1^

Association of haplotypes in genomic regions performed in French population and correlated with HOMA_IR_ and 1/S_I_ values from minimal model calculation after IVGTT.

^a^n is expressed at 2n chromosomes

^b,d^ Statistical significance in logistic regression and ANOVA, respectively

^c^ Values are expressed as mean ± SEM

*Values adjusted for hyperglycemia

•, Significance only in French population

$ P < 5 x10^-2^, OR, odds ratio; 95% CI, confidence interval; HOMA_IR_, homeostasis model assessment of insulin resistance; S_I_, insulin sensitivity; NS, non-significant

In the *SLC6A15* gene (Fig 2, lower-left panel), the associated signal spread over all 3 LD blocks with 6 significantly associated haplotypes, but only haplotype B2_H1 passed the Bonferroni, FDR and permutation tests. This haplotype together with B3_H1 and B3_H3 were significantly associated with HOMA_IR_ values (underlined in Fig 2). None of the significant independent SNPs (tested by its minor allele) was tagged in B2_H1 haplotype despite its major effect (Table 2). By contrast, 3 other haplotypes contained more than one (2 to 5) influential SNPs. This data indicates that a haplotype can reveal a signal not identified by single SNPs in statistical terms and can resume the effect of several SNPs at a time. In French population, analysis of the same blocks indicates non-significant association with S_I_ values (Table 3) suggesting that this gene does not participate to systemic IR. Finally, for the PRKCA gene (Fig 2, right-lower panel), the sliding window hit was located in block B92 (64789936 and 64809487). Among 9 associated haplotypes, two protective (B92_H6 and B93_H6) passed the Bonferroni correction test and displayed concordantly decreased HOMA_IR_ (underlined in Fig 2). These two haplotypes contained between 2 to 3 effective SNPs. Another two pathogenic haplotypes (B92_H4 and B93_H2) not sustained by Bonferroni, have been shown to be influential on HOMA_IR_ (underlined in Fig 2) and contained series of 2 to 8 independently associated SNPs (Table 2). In HAPLOVIEW, another signal was detected 103.2 kb downstream the LD block B92, between positions 64675899 (rs17762314) and 64686679 (rs7220480). Therefore, two additional blocks B65 and B66 were further mapped. In French, two rare haplotypes B65_H18 and B66_H4 (frequency 0.04 and 0.06) were associated with S_I_ values with a major effect (OR 4.43 and 2.76) as indicated in Table 3. Only B65_H18 was confirmed by Bonferroni correction. Unexpectedly, this gene was the best correlated with the cumulative criteria of MetS—for example, haplotype B92_H4 (P < 0.006; OR 3.49, 95% CI [1.43–8.52]). In summary, analysis of *in vivo* insulin sensitivity revealed that among 18 haplotypes correlated with HOMA_IR_, 6 haplotypes displayed concomitant significant variation in 1/S_I_ (indicated by “*” in Fig 2). Of note, there were haplotypes in three genes (*MUT*, *AACS* and *PRKCA*) with the highest OR and medium frequency (MAF between 0.04 to 0.28) that were the most effective in *in vivo* study while no association with 1/S_I_ was found for the transporter *SLC6A15*.

If one compares haplotype results from this study with what can be obtained only with independent SNPs on unphased DNA, these SNP markers captured the signal (P value) with at least one order of magnitude lower than the haplotypes. As for haplotypes, the set of SNPs in these regions would indicate alone the implication of these four genes. For instance, the rs2167284 in the block B3 of the *MUT* gene was associated with P < 1.27 x 10^−4^, Bonferroni P < 1.40 x 10^−2^ (see S5 Table for all SNPs). However, haplotypes detected in refined LD blocks delivered better signals and were able to better define the correlation between haplotypes among LD blocks. For the *PRKCA* gene the situation was different since SNPs had similar (P value) significance to haplotypes—for example, rs9902356 with OR of 2.12 was associated with P < 3.47 x 10^−4^ with Bonferroni P < 3.82 x 10^−2^, which is comparable to that obtained for corresponding haplotype B65_H18. Finally, the use of SNPs allowed us to replicate results (S4 Table) in all other MetS collections. Thus, among 17 associated SNPs with IR and sustained by Bonferroni or FDR tests, 13 SNPs were replicated on the extended sample (n = 832) in the Mediterranean area. Taken together, these data indicate that fine-scale haplotype mapping is complementary to independent SNP analysis and delivered more detailed genetic information at the population level.

### Association with BCAA plasma levels

**Fig 3:**
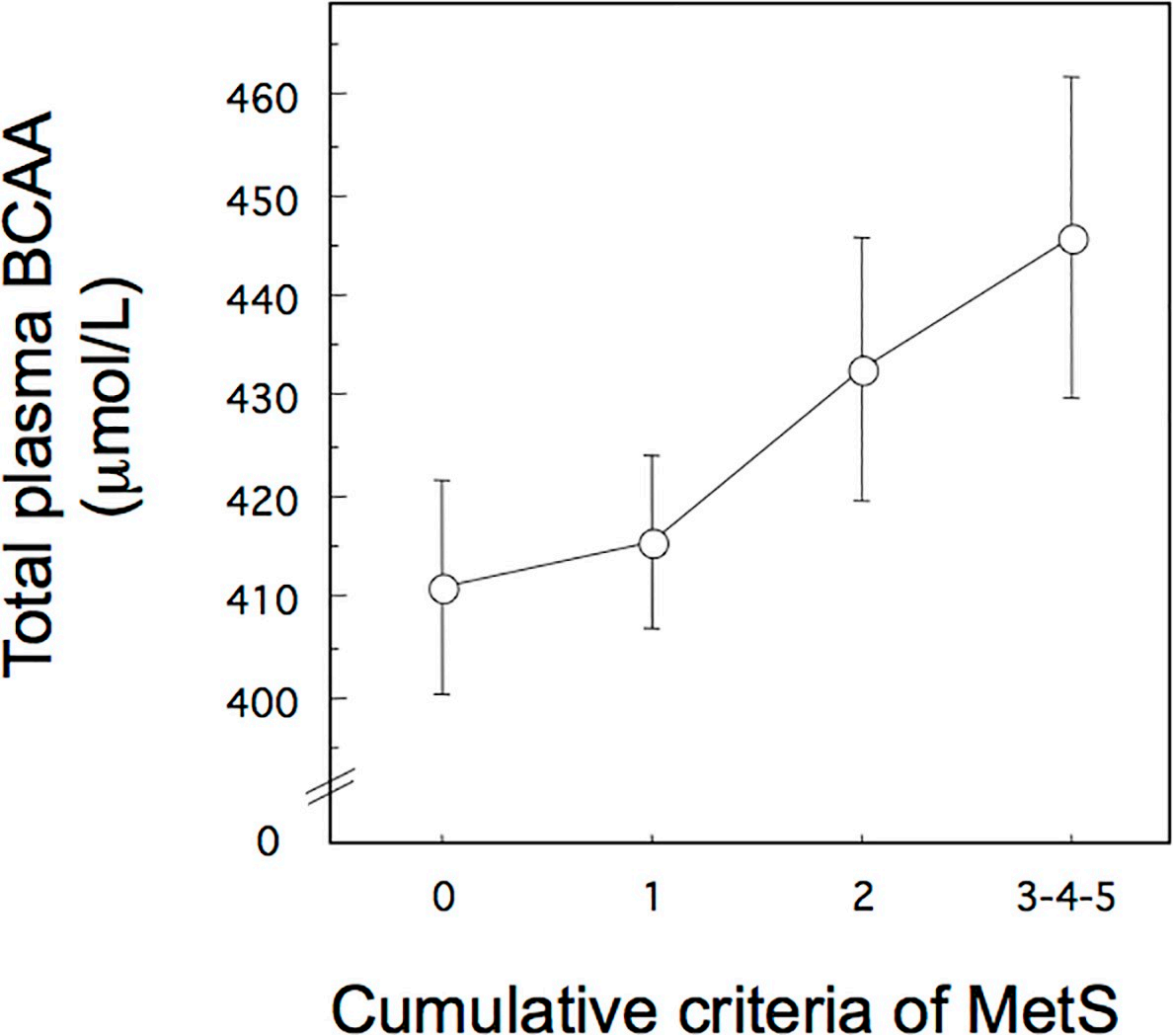
Variation of total BCAA plasma levels with cumulative criteria for MetS in French population. BCAA values represent the mean ± SE. MetS is defined with having 3 or more criteria.

The SNPs and haplotypes analysis were able to drive the attention towards four genomic regions with potential role in IR. In the next step, we explored the association of haplotypes on BCAA plasma levels. As a starting point in searching for correlation with gene defects, we explored relationship between total BCAA plasma levels and IR. As shown in Fig 3, in subgroup of French patients where data were available, there was a good correlation in ANOVA between BCAA plasma levels and cumulative criteria for MetS (P < 0.019, interaction factor α of 0.82), the correlation being more significant in females (P < 0.034, α = 0.76). Clinical and laboratory profiles of patients with MetS, including plasma BCAA levels are indicated in (S7 Table). In MetS analyzed at *2n* chromosomes, total plasma BCAA varied from 410.27 ± 7.03 (mean ± SEM, 2n = 158) to 445.71 ± 15.89 μmol/L (2n = 26) with P < 0.057 and interaction factor α = 0.46. The relationship was more significant for Leu with values from 123.16 ± 1.32 to 137.18 ± 5.66 μmol/L (P < 0.028, α = 0.58) but not for Ile and Val, although these two last amino acids displayed a trend to increased levels (P < 0.09 and 0.2, respectively). Among components of MetS, total BCAA was correlated in ANOVA with hyperglycemia (defined as nominal variable (0/1) with cut-off at 5.6 mmol/L) with P < 0.0015 (α = 0.9) and displaying values from 406.65 ± 7.21 (2n = 156) to 463.31 ± 10.56 μmol/L (2n = 28) in both men and women. There was no significant correlation between the raise in BCAA levels and the severity of IR measured by HOMA_IR_ index (P = 0.45) explained by the complex nature of HOMA_IR_, including systemic IR *per se* and inflammatory components or variation in BMI. To understand such complex interaction, predictive values of HOMA_IR_ were calculated based on BMI (in females and males separately and in each ethnic group) and then residuals of HOMA_IR_ were calculated to separate individuals with over- and under-estimated values. Total BCAA levels were well correlated with HOMA_IR_ in linear regression in over- and under-estimated individuals with r^2^ = 0.21 and 0.057, respectively (S1 Fig, left panel). In both groups, the Spearman correlation was significant with P < 0.022. In logistic regression, the nominal variable (0/1) of high BCAA levels (cutoff of 414.6 μmol/L, corresponding to the median) was associated with IR-individuals with HOMA_IR_ index higher than the value predicted by BMI, with P < 0.0007 and OR 0.19, 95% CI [0.073–0.49]. Correlation between BCAA levels and IR was also found considering the *in vivo* values of S_I_. Thus BCAA levels were correlated to S_I_ with r^2^ of 0.093 (S1 Fig, right panel) and P < 0.018 in Spearman test (P < 0.0022 in female population). Values of S_I_ were more significant for Val (P < 0.031) and less significant for Leu and Ile (P < 0.0402 and 0.07, respectively).

All haplotypes were tested in ANOVA for potential correlation with BCAA plasma levels and significant associations are indicated in S8 Table, while the summary of significant and concordant association is displayed in Table 4. Strikingly, only 43% (and 21% if one refers only to French population) of effective haplotypes on IR were also associated with increased BCAA plasma levels. Among the four genes, only *MUT*, *AACS* and *PRKCA* presented concordant effects while results for *SLC6A15* were discordant. In general, there was not a clear pattern among each amino acid (Leu, Ile and Val) and the association appeared rather common for all BCAA. Although there were numerous haplotypes specifically influential on BCAA levels (indicated by “•” in Fig 2), only several haplotypes were concordantly associated with IR and BCAA levels. For instance, in the *MUT* gene, there were 7 haplotypes correlated with BCAA, but only one B2_H12 concordantly and statistically associated on IR. The influential haplotypes on BCAA were located mainly in the LD block B2. In the *AACS*, there were 5 influential haplotypes on both BCAA and IR all over the 3 blocks. For *PRKCA*, a good correlation was found between IR and BCAA where two pathogenic haplotypes for IR (B65_H18 and B92_H1) were associated with increased levels of Leu and/or Val. If one consider the association with HOMA_IR_ index and validation by *in vivo* effects, only two haplotypes B17_H1 in *AACS* and one in *PRKCA* B65_H18 presented significant and concordant effects considering all three criteria of HOMA_IR_, *in vivo* S_I_ index for insulin sensitivity and increase in BCAA plasma level. Although IR and BCAA are definitively correlated to gene variations, fine-scale haplotype mapping captured a more diversified signal that may be used for further functional studies or clinical applications.

**Table 4:** Summary of haplotypes concordantly associated with IR and BCAA plasma levels in French population.

Haplotype ID	Sequence	Association with IR direction (+/-)^a^	Leucine		Valine		Isoleucine	
			non-carriers	carriers	non-carriers	carriers	non-carriers	carriers
***MUT***								
B2_H12	CC**GG**GACGC	- S	125.03 ± 2.48	105.87 ± 8.15*	230.17 ± 3.85	204.40 ± 12.20*	66.14 ± 1.45	56.68 ± 4.49*
***AACS***								
B17_H1	GTCA	+ S (French)	127.84 ± 7.77	142.52 ± 6.35	216.40 ± 13.11	250.54 ± 11.10	62.63 ± 3.23	75.51 ± 2.93*
B18_H3	CGTCTT	- S	126.00 ± 2.93	117.07 ± 4.24	234.15 ± 4.61	214.36 ± 6.15*	66.42 ± 1.66	62.53 ± 2.52
B18_H4	CGTTTT	+ S	119.18 ± 2.76	135.22 ± 4.68*	222.36 ± 4.28	242.58 ± 7.32*	63.78 ± 1.65	69.24 ± 2.42
B18_H6	CTTCTG	+ S (French)	123.80 ± 2.54	130.61 ± 4.47	225.28 ± 3.90	240.60 ± 7.44	64.39 ± 1.36	71.47 ± 2.76*
B19_H11	CGCT**T**GT	+ S	119.63 ± 2.72	135.02 ± 5.06*	223.48 ± 4.22	240.11 ± 7.98	63.92 ± 1.59	69.23 ± 2.91
***PRKCA***								
B65_H18	GAAGTA**C**CG	+ S	124.47 ± 2.26	137.84 ± 11.18	226.18 ± 3.50	265.82 ± 15.99*	65.53 ± 1.27	71.17 ± 4.79
B92_H1	GCGAA	+ S	127.59 ± 6.14	148.16 ± 7.82*	220.58 ± 11.23	256.16 ± 12.89*	67.65 ± 3.27	72.74 ± 3.27

Haplotypes associated with increased BCAA plasma levels (μmol/L) were evaluated in non-carriers and carriers in ANOVA. The table presents only associated haplotypes with BCAA and concordant with significant effects on IR either by HOMA_IR_ index or S_I_. The correlation for all haplotypes is indicated in S8 Table. SNPs within haplotypes underlined and in bold are those concordantly associated with IR and BCAA and analyzed in Haploreg v.4.1 (Table 5). Significant values are indicated in bold and statistical significance levels as * for P < 5 x 10^−2^.

^a^Association of each haplotype with IR (direction); significant, S; +, pathogenic; -, protective; (French) stands for significant only in French population; Independent SNPs with positive association were mapped in corresponding haplotype (underlined and bold) as following: *MUT* B2_H12 for rs753849 (G/A), rs17674678 (G/A), *AACS* B19_H11 for rs12818316 (T/C), PRKCA B65_H18 for rs9902356 (C/G).

In the hypothesis that these complex signals would be related to transcriptional activity, we analyzed associated independent SNPs using Haploreg v.4.1. For SNP analysis we followed the same strategy detecting firstly SNP associated with BCAA plasma levels in correlation trend test and then crossed with those associated with IR. Importantly, intercrossing of these two pools of SNPs gave identical results as haplotype analysis. Thus concordantly associated SNPs are those indicated in Table 4 (bold and underlined), while their prediction for alteration in transcriptional activity is indicated in Table 5. Prediction for all remaining associated SNPs and mapped in haplotypes are presented in S9 Table. For SNPs associated only with IR, 112 transcriptional factors (TFs) were noted and for those influential uniquely on BCAA there were 28 TFs annotated. Finally, for SNPs effective on both IR and BCAA levels we found concordant effects for *MUT* gene with haplotype B2_H12 containing the rs17674678, for *AACS* with haplotype B19_H11 containing rs12818316 and for *PRKCA* with haplotype B65_H18 containing the leader SNP rs9902356. For these influential SNPs we found annotated 7 TFs (CEBPA, CEBPB, p300, TCF11::MafG, GR, Pax-5 and Znf143) in haplotypes concordantly associated with IR and BCAA levels.

**Table 5:** Haplotypes associated with IR and BCAA plasma level analyzed in Haploreg v.4.1.

SNP ID	Closest gene	Associated parameter	Associated haplotype	Sequence	Regulatory motifs altered
rs17674678	*C6orf138/MUT*	IR+BCAA	B2_H12	CCG**G**GACGC	CEBPA, CEBPB, p300
rs9902356	*PRKCA* (within gene)	IR+BCAA	B65_H18	GAAGTA**C**CG	TCF11::MafG
rs12818316	*TMEM132B/AACS*	IR+BCAA	B19_H11	CGCT**T**GT	GR, Pax-5, Znf143

Transcription factors binding motifs changes are indicated for each individual SNPs. Effect of SNPs was tested by Correlation Trend Test and associated haplotypes were selected from Table 2. Annotation for all the effective SNPs within haplotypes are indicated in S9 Table.

## Discussion

In this paper, we present for the first time evidence for the association with IR of four genomic regions close to genes involved in BCAA catabolism or its regulation. The *MUT* gene operates in the distal steps of BCAA degradation, while *AACS*, albeit included in the KEGG database, is involved in ketone bodies metabolism. The *SLC6A15* gene encodes for a specific BCAA transporter operating in the brain and under the regulation of *PRKCA*. The association of these genes with IR was not previously reported and none of corresponding effective SNPs were reported as being associated with IR. These findings reiterate the potential relationship between IR and BCAA metabolism and add stronger evidence for common genetic susceptibility, although the causality relationship cannot be established from these studies. As strategic point, haplotype mapping was an alternative way to identify association signals compared with independent SNPs. Our data indicate that this strategy delivers more detailed information not captured by SNPs at the population level. Although, haplotypes and mapped SNPs within haplotypes suggest a dissociation of the signal between BCAA and IR, at least three haplotypes were concordantly associated to *in vivo* IR and BCAA plasma levels. Taken together these data may help understanding of the relationship between IR and BCAA metabolism and the development at clinical scale of genetic markers in complex conditions such as MetS.

To efficiently and directly define the most significant regions from the gene panel, we first used the sliding window procedure for short 4-SNP haplotypes. Larger haplotypes were then obtained on PHASE 2.1 as a function of refined LD blocks in HAPLOVIEW program. We limited our investigation to the most 4 significant hits, although we cannot exclude the involvement of other genes with lower association, particularly those previously identified in T2D. In our sample *BCAT*, *BCKDH* and *PPM1K* genes displayed signals with P values that were at least one order of magnitude lower. For instance, the two leader SNPs (rs1440581 and rs7678928) for *PPM1K* previously described in GWAS [24] were associated in our sample at P = 0.25. Further studies at higher density (10,000 genome project) or ultra-dense sequencing would be necessary to better prioritize these genes. It is also possible that patients with IR in the context of MetS explored in this study were in some way different from patients with T2D in previously genetic studies [24]. Nevertheless, our data suggest that systemic IR defined by HOMA_IR_ index alone may be heterogeneous in nature and explained by different mechanisms, including insulin action, inflammation, insulin secretion or other complex processes leading to arterial hypertension. Along this line, it should be noted that *SLC6A15* gene was not correlated with *in vivo* systemic IR suggesting perhaps effects uniquely at the central nervous system.

In sliding window procedure the Bonferroni correction was not applied over 29,087 SNPs. However, to properly compare haplotype-mapping with canonical use of independent SNPs, we tested the association of 104 corresponding SNPs to the genomic regions. For the same regions, haplotype-mapping indicated 11 positive hits supported by Bonferroni correction, which was at one order of magnitude higher than independent SNPs, indicating that association signal was better captured by haplotypes. Although none of the SNPs display GWAS significance (10^−8^), 7 SNP markers passed Bonferroni test and were replicated over the extended Mediterranean sample. In the replication test, the inflation factor λ for the genomic control method was 1.3. This relatively high value may be explained either by population stratification or by the polygenic nature of IR involving several genes. Data presented in Table 2 represent genuine associations without adjustment for BMI. However, to properly study the effect of BMI, we examined the residuals after correction of HOMA_IR_ values by BMI separately in two different ethnic populations and as function of gender, procedure well established as previously shown [41]. The same strategy was used for analysis of BCAA plasma levels. In this study, there was not a significant difference between MetS and controls subjects for BCAA plasma levels with exception of Leu calculated at 1n or 2n chromosomes. This result suggests that measurement of total BCAA plasma levels would not be sufficient as a biomarker. Recent studies indicated that plasma BCAA concentrations might not accurately reflect the BCAA kinetics. Indeed, in both human and murine models, better correlations can be found between IR and BCAA kinetics [44, 45]. Further studies are however necessary to understand the independent effect of haplotypes of candidate genes. Results from our investigation indicated that haplotype effects were consistent with static levels of BCAA and independent from BMI.

In this study we identified four genomic regions close to annotated genes in BCAA metabolism. The SNPs and respective haplotypes in these regions are novel and were not previously described in GWAS or NCBI (PUBMED) with some exceptions (see later). How these regions would regulate the function of annotated genes remains unknown but very likely they are regulatory regions. As a whole, our data drive the attention for further biological investigation in regulation of gene expression.

The *MUT* (*methylmalonyl-CoA mutase*) gene encodes for a mitochondrial enzyme catalyzing isomerization of methylmalonyl-CoA to succinyl-CoA (MIM 609058). This gene is the only one indeed involved in BCAA metabolism and degradation. Since *MUT* mutations induce MMA and some rare cases were described with severe IR [23], we cannot exclude potential concomitant mutations in the coding region, but based on clinical profile, this seems unlikely. Interestingly, another report indicates that a specific haplotype (CAAAAAAAGTGACTTCGCTTC) downstream our region was frequently associated with Mendelian mutations in Hispanics (positions 49,368,844–49,434,931) [20]. This haplotype was not found in our sample. However, a slightly modified haplotype at 2 positions (GAAAAATTGTGACTACCCTAG) was detected in our sample but unrelated with IR (P-value < 0.38 in χ^2^ HAPLOVIEW). How *MUT* contributes to systemic IR is not completely understood. Previous elegant studies in humans and rodents suggested alterations of the lipid metabolism [19]. Other studies have indicated the role of proprionate in the expression of *MUT* gene involved in its metabolization. Both proprionate and succinate serve as substrate for intestinal gluconeogenesis, which is sensed by nervous system surrounding the portal vein [46, 47]. Therefore, MUT gene, although involved in systemic IR, may also imply regulatory mechanisms by the central nervous system.

In the *MUT* region, two haplotypes (B3_H12 and B4_H2) were concordantly associated with IR and *in vivo* insulin sensitivity, but their frequencies (0.08 and 0.07, respectively) suggest that they are low-frequent variants but with major effects, situation which is typical for complex conditions. Comprehensive review of all effects described in this study, indicate that *MUT* gene would be influential concordantly on both BCAA and IR by the haplotypes B2_H12 (frequency 0.12) located in block B2. The effect of this haplotype was synergistic to haplotypes in the next blocks (B3_H3 and B4_H8 with frequency of 0.22) covering at least 10% of the population (Fig 2).

The second genomic region was located close to *AACS* (*acetoacetyl-CoA synthetase*) on Chr 12q24.31. The gene was included in this study because of its annotation in the KEGG database for BCAA metabolism, but the enzyme encoded by this gene is a ketone body-utilizing ligase (EC 6.2.1.16) with a role in lipid synthesis through the non-oxydative pathway (MIM 614364). The enzyme converts acetoacetate into its CoA ester in both hepatic and extrahepatic tissues (e.g., brain) and its activity explains the effect of hypocholesterolemic agents [48]. In humans, its activity is crucial since ketone bodies (e.g., acetoacetate and β-hydroxybutyrate) are sources of energy for the brain in the absence of glucose (e.g., starvation) [49]. As for the MUT gene, the relationship with IR is not completely understood but *AACS* is said to be involved in the metabolic flux of BCAA (e.g., Leu) at a distal site. In this study we identified in the region of *AACS* several haplotypes with concordant modifications in IR and BCAA, although the causality relationship cannot be established and no clear pattern was detected for individual amino acids. Although the population size was not sufficient to demonstrate gender dimorphism in statistical terms, the dimorphism observed for AACS for all three amino acids, fits well with the high expression of *AACS* in males under the effect of testosterone [49].

The third region was located downstream of the *SLC6A15* (*solute carrier family 6*, *neurotransmitter transporter*, *member 15*) gene, which is also known as a *sodium-coupled branched-chain amino acid transporter* (*SBAT1*) (MIM 607971). The gene encodes (Chr 12q21.31) for an amino-acid transport protein with strong preference for BCAA and methionine [50]. In this gene, only one haplotype was significantly associated with IR (B2_H1) and concordant with high HOMA_IR_ (P < 0.0156). However, when we searched for correlation with BCAA plasma levels, all haplotypes displayed discordant association and therefore this gene was not included in the summary in Table 4. The non-significant association with IR defined by *in vivo* S_I_ levels, suggests that this gene poorly participates to systemic IR, and therefore it is likely that *SLC6A15* exerts its effect by a different mechanism. In humans, the *SLC6A15* gene was previously involved in depression [51]. Thus, a GWAS performed in German, Dutch and African Americans identified rs1545843 by the A allele (position 84564068) as a marker for depression [52]. This SNP was located at 287 kb distal from the gene and just before our LD block B1. Interestingly, the A allele of rs1545843 was correlated with IR in our sample. Thus, the alternative G allele was associated with P < 0.0022 and OR 0.63 95%CI [0.47–0.85]. From previous reports, another two SNPs, one intragenic (rs17183577) and one upstream (rs7980296) were associated with IR in boys and young adults in a Swedish population [50]. This rs7980296 was obtained by imputation in our sample at 735 kb upstream of the studied region, suggesting that the association signal may be widespread over a larger genomic region.

The forth region studied contains the *PRKCA*, an immediate regulator of *SLC6A15* in nervous tissue [53]. Indeed, the *PRKC*-*alpha* (*PRKCA*) is activated in brain tissues and was found in human neuroblastoma cells [54]. Since *PRKCA* was shown to regulate the *SLC6A15* gene product, our data may be suggestive for a potential effect at the central nervous system. This gene is very likely involved in numerous cellular processes at both systemic and central nervous system. In our study, all *PRKCA* haplotypes effective on IR were also effective and concordant on BCAA (Table 4). Interestingly, several SNPs in the same region were reported as being associated either with alcohol dependence (rs10603061) or multiple sclerosis (rs1010546) suggesting that the region described here may be regulatory in nature and thus reinforcing a potential brain mechanism in regulation of amino acids metabolism [55].

This study is limited by the size of the population at the discovery stage, involving only two ethnic populations. Extension of our study over other Mediterranean populations available on MEDIGENE program would considerably complicate haplotype mapping. Another limitation was the unavailability of a metabolomics profile that would better characterize potential gene defects by measuring metabolites at various steps of BCAA metabolism. Despite these limitations, implication of these genomic regions in IR is novel since none of SNPs described here were previously reported in GWAS of other complex diseases. Thus, our study drives the attention towards these potentially regulatory regions that warrant further biological investigation in relation with IR. The *PPM1K* gene identified by MR was not associated with IR in our study. Therefore, we cannot exclude the possibility that our collection of MetS contains subjects with IR different from T2D as in previous GWAS. Further studies at epidemiological level would be necessary to compare these two conditions. In conclusion, data presented here shed new light on the complex mechanisms that would explain the pathogenesis of IR in relation to BCAA metabolism and its regulation without establishing causality and highlight the importance of detecting association signals in phased DNA as solid strategy for designing genetic markers at clinical scales in ethnic populations.

## Supporting information

S1 Fig Correlation between total plasma BCAA levels and insulin resistance in French population. Left panel, correlation between BCAA levels and HOMA_IR_ index of insulin resistance. Right panel, correlation between BCAA levels and insulin sensitivity index S_I_. Predicted HOMA_IR_ index values were calculated from body mass index (BMI) and residuals indicated over- and under-underestimated values. Circles represent the sub-population with under-estimated values and squares with over-estimated values.
(TIFF)

S1 Table Gene candidates (n = 79) for the study of BCAA metabolism in MetS. After exclusion, there were 73 genes included in the genetic study. Genes absent from MEDISCOPE GeneChip and excluded from the study are indicated in grey; Genes for which imputation procedure failed are indicated in bold
(PDF)

S2 Table Phenotype features of patients with and without insulin resistance for genetic study. ^a^Controls and IR groups were compared using Mann-Whitney test for numerical variable and χ2 for nominal variables; ^b^In defining hyperglycemia, hypertension, high triglycerides and low HDL nominal variable, treatment of pre-diagnosed type 2 diabetes, high blood pressure or dyslipidemia were also considered; ^c^Insulin resistance was considered as function of HOMA_IR_ values; ^d^Obesity was considered based on BMI > 30 kg/m^2^; IR, insulin resistant; NA, non-applicable; NS non-significant; Values are expressed as mean ± SEM.
(PDF)

S3 Table The most significant short (4 SNPs) haplotypes associated with insulin resistance from sliding window. The sliding window was applied to imputed SNPs in French and Romanian populations using a windowing of 4 SNPs. Positive associated haplotypes were classified by P-significant value (Bonferroni correction was not applied). The most significant hits (P ≤ 9.39 x 10^−5^) were retained for further higher length haplotype mapping or independent SNP analysis. Haplotypes were also tested against MetS but the unique positive hit found upstream IGF1 gene was not associated with IR.
Chr, chromosome; OR, odds ratio; 95% CI, confidence interval; X, stands for large insertion CACACTGATTTCAGGGGGGTCT; *Short haplotypes in IGF-1 gene were associated only with MetS but not with IR; **Note that the region indicated containing the initial hit was extended in the following LD block used in haplotype mapping in HAPLOVIEW.
(PDF)

S4 Table The most significant associated SNPs with IR in French and Romanians and replication extended to other Mediterranean populations. Genomic regions were identified by sliding window and corresponding SNPs were tested independently for association with IR. P-values of all 104 SNPs are indicated in S5 Table.
^a^The closest upstream or downstream genes are shown for intergenic SNPs; ^b^Tested allele; ^c^Significance by logistic regression using basic model; ^d^For replication, SNPs were tested in the extended sample (n = 832) and P-value (χ^2^) was corrected by inflation factor λ = 1.3; *P < 0.05 using 10k permutations in HAPLOVIEW; ^$^Another 8 SNPs were in linkage disequilibrium with r^2^ values of 1 (rs2403184, rs1384320, rs1384321), 0.99 (rs1482441, rs10862831, rs10779081) and 0.98 (rs10779083, rs1482429) in Haploreg v4.1 and with the same association values.
(PDF)

S5 Table Association of 104 SNPs with IR in four genomic regions in French and Romanians. Significant SNPs after Bonferroni and/or FDR corrections are indicated in S4 Table.
^a^For intergenic SNPs, the closest upstream or downstream genes are indicated; ^b^Tested allele; ^c^Statistical significance assessed by logistic regression using basic model; Chr, chromosome; MAF, minor allele frequency; G/I, genotyped/imputed; FDR, false discovery rate; OR, odds ratio; 95% CI, confidence interval; NS, non-significant.
(PDF)

S6 Table Reconstruction of haplotypes with PHASE 2.1 program and association (χ^2^ test) with insulin resistance from HAPLOVIEW. Genomic regions are indicated in the text and the LD blocking structure is depicted in Fig 2. Only frequent (> 0.1) and significant associated haplotypes are indicated in Table 2.
(PDF)

S7 Table Clinical and laboratory features of MetS and control subjects in French population. Data are presented as mean ± SEM. Controls and MetS groups were compared using Mann-Whitney test for numerical variable and χ^2^ for nominal variable.
a, NA stands for non applicable, NS stands for non-significant; b, In defining hyperglycemia, hypertension, high triglycerides and low HDL nominal variable, treatment of pre-diagnosed type 2 diabetes, high blood pressure or dyslipidemia were also considered; c, Insulin resistance was considered as function of HOMA_IR_ values; d, Obesity was considered based on Body Mass Index (BMI) > 30 kg/m^2^; e, Plasma BCAA levels were available only for 92 subjects.
(DOCX)

S8 Table Association of haplotypes with BCAA plasma levels in French population. BCAA plasma levels (μmol/L) were evaluated in French population between non-carriers and carriers in ANOVA. Significant values are indicated in bold and statistical significance levels as * for P < 5 x 10^−2^ and ** for P < 5 x 10^−3^.
^a^Association of each haplotype with IR (direction) significant (S) or non-significant (NS): +, pathogenic; -, protective; (French) stands for significant only in French population; Independent SNPs with positive association were mapped in corresponding haplotype (underlined and bold) as following: MUT B2_H1 for rs12527508 (A/G), B2_H4 for rs12527508 (A/G), B2_H6 for rs12527508 (A/G), rs62408552 (G/A), rs2503674 (T/C), B2_H12 for rs753849 (G/A), rs17674678 (G/A), B2_H13 for rs1199183 (T/C), rs753849 (G/A), B3_H2 for rs325337 (G/A), *AACS* B19_H11 for rs12818316 (T/C), *SLC6A15* B1_H2 for rs79588760 (T/C), B1_H4 for rs732438 (T/C), B2_H7 for rs7301137 (C/T), B3_H2 for rs79558964 (C/T), *PRKCA* B65_H6 for rs10491203 (G/A), rs8071795 (C/T), B65_H18 for rs9902356 (C/G), B66_H9 for rs8070556 (T/G), rs1010546 (T/C), rs16960016 (T/C), rs8072511 (G/C).
(DOCX)

S9 Table Haploreg analysis of SNPs effective on IR and/or BCAA. Transcription factor binding motifs changes are indicated for each individual SNPs associated with IR (S5 Table) and/or individual BCAA plasma level (Correlation Trend Test). SNPs are indicated in corresponding haplotypes effective on IR and/or BCAA as shown in Tables 2–4.
(DOCX)
