# Supplementary figures and images for: Fine-scale haplotype mapping of MUT, AACS, SLC6A15 and PRKCA genes indicates association with insulin resistance of metabolic syndrome and relationship with branched chain amino acid metabolism or regulation
Source: PLoS One. 2019 Mar 26;14(3):e0214122. doi: 10.1371/journal.pone.0214122 (PMC6435171; doi:10.1371/journal.pone.0214122)

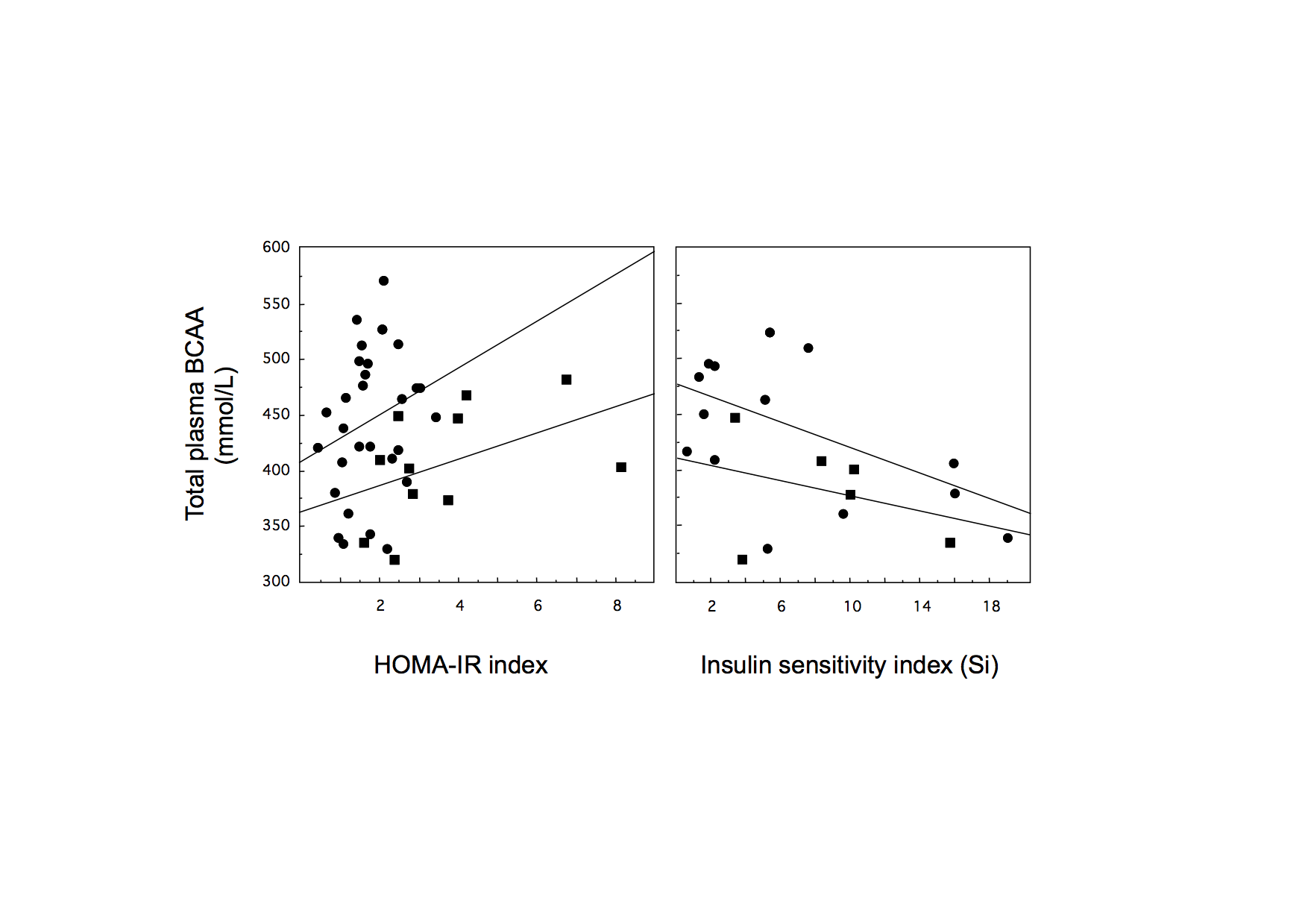

Supplement: S1 Fig — Left panel, correlation between BCAA levels and HOMAIR index of insulin resistance. Right panel, correlation between BCAA levels and insulin sensitivity index SI. Predicted HOMAIR index values were calculated from body mass index (BMI) and residuals indicated over- and under-underestimated values. Circles represent the sub-population with under-estimated values and squares with over-estimated values. (TIFF) [file pone.0214122.s001.tiff]
