# Supplementary material for: Fine-scale haplotype mapping of MUT, AACS, SLC6A15 and PRKCA genes indicates association with insulin resistance of metabolic syndrome and relationship with branched chain amino acid metabolism or regulation
Source: PLoS One. 2019 Mar 26;14(3):e0214122. doi: 10.1371/journal.pone.0214122 (PMC6435171; doi:10.1371/journal.pone.0214122)
Supplement: S1 Table — After exclusion, there were 73 genes included in the genetic study. Genes absent from MEDISCOPE GeneChip and excluded from the study are indicated in grey; Genes for which imputation procedure failed are indicated in bold (PDF) [file pone.0214122.s002.pdf]

| | | | | | |
|---|---|---|---|---|---|
| **Genes of BCAA catabolism from KEGG database** | | | | | |
| ACADM | ALDH9A1 | DBT | HMGCL | HMGCS2 | OXCT2 |
| AOX1 | **HADHA** | HADHB | HIBCH | MCEE | ACAA1 |
| EHHADH | MCCC1 | PCCB | HADH | AGXT2 | ALDH7A1 |
| **HMGCS1** | MCCC2 | OXCT1 | ACAT2 | BCKDHB | HMGCLL1 |
| MUT | DLD | HIBADH | ALDH1B1 | AUH | ACADSB |
| ACAD8 | ACAT1 | AACS | ACADS | ALDH2 | BCAT1 |
| PCCA | ALDH6A1 | IVD | ABAT | **ACSF3** | ALDH3A2 |
| ACAA2 | BCAT2 | BCKDHA | IL4I1 | HSD17B10 | ECHS1 |
| **Genes involved in BCAA transport** | | | | | |
| SLC3A2 | SLC6A15 | SLC7A7 | SLC7A5 (LAT1) | SLC7A6 | SLC43A2 |
| SLC1A5 | SLC7A8 (LAT2) | | | | |
| **Gene involved in BCAA regulation or influential on BCAA plasma level** | | | | | |
| GCKR | TRIM63 | ABCD3 | SCP2 | PRKCE | PDK1 |
| KLF15 | ACOX2 | PDK4 | FBXO32 | IGF1 | ABCD2 |
| ACAD10 | TRMT61A | ABCD4 | PRC1 | **DDX19A** | CBLN1 |
| ABCA10 | PRKCA | ABCD1 | PPM1K | BCKDK | |
