# Supplementary material for: Fine-scale haplotype mapping of MUT, AACS, SLC6A15 and PRKCA genes indicates association with insulin resistance of metabolic syndrome and relationship with branched chain amino acid metabolism or regulation
Source: PLoS One. 2019 Mar 26;14(3):e0214122. doi: 10.1371/journal.pone.0214122 (PMC6435171; doi:10.1371/journal.pone.0214122)
Supplement: S2 Table — aControls and IR groups were compared using Mann-Whitney test for numerical variable and χ2 for nominal variables; bIn defining hyperglycemia, hypertension, high triglycerides and low HDL nominal variable, treatment of pre-diagnosed type 2 diabetes, high blood pressure or dyslipidemia were also considered; cInsulin resistance was considered as function of HOMAIR values; dObesity was considered based on BMI > 30 kg/m2; IR, insulin resistant; NA, non-applicable; NS non-significant; Values are expressed as mean ± SEM. (PDF) [file pone.0214122.s003.pdf]

	Controls<br>Non-IR	Cases<br>IR[c]	P value[a]
n	338	127	NA
Gender (Females/Males)	308/30	108/19	NS
Age (years)	44.49 ± 1.25	49.69 ± 1.15	< 0.0034
BMI (kg/m^2)	25.21 ± 0.36	34.95 ± 0.69	< 0.0001
Waist (cm)	90.84 ± 1.41	107.55 ± 1.57	< 0.0001
Fasting Glucose (mmol/L)	4.93 ± 0.09	6.20 ± 1.19	< 0.0001
Fasting insulin (mU/mL)	7.77 ± 0.89	20.20 ± 1.18	< 0.0001
Hyperglycemia (%)[b]	12.13	57.48	< 0.0001
$HOMA_{IR}$	1.67 ± 0.18	5.24 ± 0.30	< 0.0001
SBP (mmHg)	124.56 ± 2.20	137.71 ± 2.10	< 0.0001
DBP (mmHg)	76.55 ± 1.51	81.99 ± 1.37	<0.0087
Triglycerides (mmol/L)	1.20 ± 0.06	1.89 ± 0.11	< 0.0001
HDL-cholesterol (mmol/L)	1.36 ± 0.04	1.23 ± 0.03	< 0.011
Obesity (%)[d]	19.8	75.59	< 0.0001
Hypertension (%)[b]	38.46	77.95	< 0.0001
High Triglycerides (%)[b]	24.85	57.48	< 0.0001
Low HDL (%)[b]	14.49	49.60	< 0.0001
