# Supplementary material for: Fine-scale haplotype mapping of MUT, AACS, SLC6A15 and PRKCA genes indicates association with insulin resistance of metabolic syndrome and relationship with branched chain amino acid metabolism or regulation
Source: PLoS One. 2019 Mar 26;14(3):e0214122. doi: 10.1371/journal.pone.0214122 (PMC6435171; doi:10.1371/journal.pone.0214122)
Supplement: S3 Table — The sliding window was applied to imputed SNPs in French and Romanian populations using a windowing of 4 SNPs. Positive associated haplotypes were classified by P-significant value (Bonferroni correction was not applied). The most significant hits (P ≤ 9.39 x 10^−5^) were retained for further higher length haplotype mapping or independent SNP analysis. Haplotypes were also tested against MetS but the unique positive hit found upstream IGF1 gene was not associated with IR. Chr, chromosome; OR, odds ratio; 95% CI, confidence interval; X, stands for large insertion CACACTGATTTCAGGGGGGTCT; *Short haplotypes in IGF-1 gene were associated only with MetS but not with IR; **Note that the region indicated containing the initial hit was extended in the following LD block used in haplotype mapping in HAPLOVIEW. (PDF) [file pone.0214122.s004.pdf]

Chr	Position of the first SNP	Haplotype	Frequency		P-value	OR	95% CI		Location
			cases	controls			lower	upper	
12	84570410	GGAA	0.56	0.41	3.73×10^{-5}	1.80	1.35	2.41	Downstream SCL6A15
12	84570986	GAAG	0.56	0.41	4.32×10^{-5}	1.79	1.34	2.40	Downstream SCL6A15
17	64786516	X,G,A,A	0.01	0.05	4.49×10^{-5}	0.11	0.02	0.56	PRKCA
17	64789936	GAAC	0.01	0.05	4.52×10^{-5}	0.11	0.02	0.56	PRKCA
12	125651199	TATT	0.37	0.51	4.55×10^{-5}	0.54	0.40	0.73	Downstream AACS**
12	125650120	TTAT	0.37	0.51	4.55×10^{-5}	0.54	0.40	0.73	Downstream AACS
12	125656263	CACT	0.41	0.56	4.93×10^{-5}	0.55	0.41	0.74	Downstream AACS
12	125651631	TTCA	0.37	0.52	5.40×10^{-5}	0.55	0.41	0.74	Downstream AACS
12	125652337	TACC	0.37	0.52	5.40×10^{-5}	0.55	0.41	0.74	Downstream AACS
12	125651442	ATTC	0.37	0.52	5.40×10^{-5}	0.55	0.41	0.73	Downstream AACS
12	125653091	ACCT	0.37	0.52	5.40×10^{-5}	0.55	0.41	0.74	Downstream AACS
17	64790434	AACA	0.01	0.05	5.61×10^{-5}	0.11	0.02	0.55	PRKCA
12	125648159	TGGC	0.37	0.52	6.16×10^{-5}	0.55	0.41	0.74	Downstream AACS
12	125647392	ACTC	0.37	0.52	6.16×10^{-5}	0.55	0.41	0.74	Downstream AACS
12	125646451	CACT	0.37	0.52	6.28×10^{-5}	0.55	0.41	0.74	Downstream AACS
6	48282957	CATG	0.13	0.25	6.29×10^{-5}	0.46	0.31	0.69	Downstream MUT
6	48280842	CCAT	0.13	0.24	7.05×10^{-5}	0.46	0.31	0.69	Downstream MUT
6	48287802	ATGT	0.13	0.25	7.10×10^{-5}	0.46	0.31	0.69	Downstream MUT
12	103149066	CGCC	0.32	0.45	7.80×10^{-5}	0.58	0.44	0.76	Upstream IGF1*
12	103148974	CCGC	0.32	0.45	7.80×10^{-5}	0.58	0.44	0.76	Upstream IGF1*
6	48288218	TGTC	0.13	0.25	8.48×10^{-5}	0.46	0.31	0.70	Downstream MUT
6	48329893	CCGA	0.14	0.25	9.39×10^{-5}	0.46	0.31	0.70	Downstream MUT
16	8770320	GAAC	0.13	0.24	9.39×10^{-5}	0.47	0.31	0.70	ABAT
