# Supplementary material for: Fine-scale haplotype mapping of MUT, AACS, SLC6A15 and PRKCA genes indicates association with insulin resistance of metabolic syndrome and relationship with branched chain amino acid metabolism or regulation
Source: PLoS One. 2019 Mar 26;14(3):e0214122. doi: 10.1371/journal.pone.0214122 (PMC6435171; doi:10.1371/journal.pone.0214122)
Supplement: S4 Table — Genomic regions were identified by sliding window and corresponding SNPs were tested independently for association with IR. P-values of all 104 SNPs are indicated in S5 Table. aThe closest upstream or downstream genes are shown for intergenic SNPs; bTested allele; cSignificance by logistic regression using basic model; dFor replication, SNPs were tested in the extended sample (n = 832) and P-value (χ2) was corrected by inflation factor λ = 1.3; *P < 0.05 using 10k permutations in HAPLOVIEW; $Another 8 SNPs were in linkage disequilibrium with r2 values of 1 (rs2403184, rs1384320, rs1384321), 0.99 (rs1482441, rs10862831, rs10779081) and 0.98 (rs10779083, rs1482429) in Haploreg v4.1 and with the same association values. (PDF) [file pone.0214122.s005.pdf]

SNP ID	Position	Chr	Closest gene[a]	Minor/Major allele		MAF	G/I	P-value Regression[c]	Bonferroni	FDR	Replication P-value[d]	OR	95% CI	
													lower	upper
rs17674678	48174498	6	C6orf138/MUT	G	A	0.13	G	2.23×10^{-3}	NS	2.21×10^{-2}	3.18×10^{-2}	0.47	0.28	0.79
rs2167284*	48288218	6	C6orf138/MUT	T	C	0.22	G	1.27×10^{-4}	1.40×10^{-2}	1.39×10^{-2}	2.89×10^{-2}	0.47	0.32	0.71
rs325041	48329893	6	C6orf138/MUT	C	A	0.35	G	6.73×10^{-3}	NS	3.33×10^{-2}	9.54×10^{-2}	0.65	0.47	0.89
rs17762314	64675899	17	PRKCA (within gene)	G	A	0.30	I	4.36×10^{-3}	NS	2.26×10^{-2}	4.34×10^{-1}	1.57	1.15	2.13
rs2052193	64677193	17	PRKCA (within gene)	T	A	0.46	I	8.71×10^{-3}	NS	4.13×10^{-2}	9.14×10^{-1}	0.68	0.50	0.91
rs9902356*	64679742	17	PRKCA (within gene)	C	G	0.12	I	3.47×10^{-4}	3.82×10^{-2}	9.46×10^{-3}	1.44×10^{-2}	2.12	1.42	3.17
rs78518692*	64808034	17	PRKCA	A	G	0.06	G	3.27×10^{-4}	3.60×10^{-2}	1.19×10^{-2}	1.44×10^{-2}	0.24	0.10	0.61
rs4791033*	64810389	17	PRKCA	A	G	0.06	I	1.46×10^{-4}	1.61×10^{-2}	7.96×10^{-3}	1.06×10^{-2}	0.20	0.07	0.56
rs118009757	64814470	17	PRKCA	A	G	0.05	I	4.33×10^{-4}	4.77×10^{-2}	6.75×10^{-3}	1.81×10^{-2}	0.22	0.08	0.62
rs28450079	64815228	17	PRKCA	T	C	0.05	I	4.33×10^{-4}	4.77×10^{-2}	6.75×10^{-3}	1.81×10^{-2}	0.22	0.08	0.62
rs2362711	64815699	17	PRKCA	T	G	0.05	I	4.33×10^{-4}	4.77×10^{-2}	6.75×10^{-3}	1.81×10^{-2}	0.22	0.08	0.62
rs2403183*$	84567611	12	SLC6A15	G	C	0.50	I	2.24×10^{-3}	NS	1.22×10^{-2}	3.58×10^{-3}	0.64	0.48	0.85
rs73233312	125685471	12	AACS	T	C	0.06	G	8.86×10^{-4}	NS	1.07×10^{-2}	2.55×10^{-2}	0.26	0.10	0.67
rs4442602*	125710951	12	AACS	T	C	0.29	G	1.32×10^{-3}	NS	1.44×10^{-2}	6.20×10^{-3}	1.66	1.22	2.25
rs10846850*	125715717	12	AACS	T	C	0.13	G	7.65×10^{-4}	NS	1.04×10^{-2}	1.34×10^{-2}	0.43	0.26	0.73
rs12818316	125737328	12	TMEM132B/AACS	T	C	0.27	G	1.00×10^{-2}	NS	4.54×10^{-2}	4.18×10^{-2}	1.52	1.11	2.08
rs61943077	125746191	12	TMEM132B/AACS	A	G	0.36	G	1.10×10^{-2}	NS	4.79×10^{-2}	5.10×10^{-2}	0.67	0.49	0.92
