# Supplementary material for: Fine-scale haplotype mapping of MUT, AACS, SLC6A15 and PRKCA genes indicates association with insulin resistance of metabolic syndrome and relationship with branched chain amino acid metabolism or regulation
Source: PLoS One. 2019 Mar 26;14(3):e0214122. doi: 10.1371/journal.pone.0214122 (PMC6435171; doi:10.1371/journal.pone.0214122)
Supplement: S5 Table — Significant SNPs after Bonferroni and/or FDR corrections are indicated in S4 Table. aFor intergenic SNPs, the closest upstream or downstream genes are indicated; bTested allele; cStatistical significance assessed by logistic regression using basic model; Chr, chromosome; MAF, minor allele frequency; G/I, genotyped/imputed; FDR, false discovery rate; OR, odds ratio; 95% CI, confidence interval; NS, non-significant. (PDF) [file pone.0214122.s006.pdf]

SNP ID	Position	Chr	Closest gene[a]	Minor allele[b]	Major allele	MAF	G/I	P-value regression[c]	Bonferroni	FDR	OR	95% CI	
												lower	upper
rs1199183	48152123	6	C6orf138/MUT	T	C	0.25	G	1.73×10^{-1}	NS	NS	1.26	0.91	1.74
rs10948402	48156101	6	C6orf138/MUT	A	C	0.29	G	9.90×10^{-1}	NS	NS	1.00	0.73	1.38
rs753849	48160865	6	C6orf138/MUT	G	A	0.43	G	9.92×10^{-1}	NS	NS	1.00	0.75	1.34
rs17674678	48174498	6	C6orf138/MUT	G	A	0.13	G	2.23×10^{-3}	NS	2.21×10^{-2}	0.47	0.28	0.79
rs12527508	48195385	6	C6orf138/MUT	A	G	0.43	G	7.22×10^{-2}	NS	NS	0.76	0.57	1.03
rs62408552	48205578	6	C6orf138/MUT	G	A	0.11	G	1.35×10^{-1}	NS	NS	0.68	0.41	1.14
rs2503674	48228338	6	C6orf138/MUT	T	C	0.08	G	4.21×10^{-2}	NS	NS	0.53	0.28	1.01
rs79741232	48230854	6	C6orf138/MUT	A	G	0.03	G	9.59×10^{-1}	NS	NS	0.98	0.41	2.35
rs12208936	48280842	6	C6orf138/MUT	T	C	0.09	G	4.27×10^{-2}	NS	NS	1.63	1.03	2.58
rs616178	48282957	6	C6orf138/MUT	A	C	0.08	G	2.11×10^{-1}	NS	NS	1.40	0.83	2.34
rs689261	48287802	6	C6orf138/MUT	G	A	0.50	G	8.91×10^{-2}	NS	NS	1.28	0.96	1.72
rs2167284	48288218	6	C6orf138/MUT	T	C	0.22	G	1.27×10^{-4}	1.40×10^{-2}	1.39×10^{-2}	0.47	0.32	0.71
rs525511	48289027	6	C6orf138/MUT	T	G	0.29	G	9.96×10^{-2}	NS	NS	1.30	0.95	1.78
rs1889757	48289514	6	C6orf138/MUT	C	T	0.11	G	8.06×10^{-2}	NS	NS	1.48	0.96	2.28
rs2396859	48300857	6	C6orf138/MUT	T	C	0.27	G	9.37×10^{-1}	NS	NS	1.01	0.73	1.40
rs325337	48321824	6	C6orf138/MUT	G	A	0.23	G	1.52×10^{-1}	NS	NS	1.28	0.92	1.78
rs76185631	48322656	6	C6orf138/MUT	T	C	0.08	G	7.70×10^{-1}	NS	NS	0.92	0.54	1.59
rs325041	48329893	6	C6orf138/MUT	C	A	0.35	G	6.73×10^{-3}	NS	3.33×10^{-2}	0.65	0.47	0.89
rs6458607	48346488	6	C6orf138/MUT	T	C	0.15	G	3.93×10^{-1}	NS	NS	1.19	0.80	1.78
rs35163082	48357970	6	C6orf138/MUT	A	G	0.02	G	2.94×10^{-2}	NS	NS	0.17	0.02	1.33
rs114337315	48364413	6	C6orf138/MUT	G	A	0.06	G	4.05×10^{-1}	NS	NS	1.29	0.71	2.35
rs6941242	48371928	6	C6orf138/MUT	T	C	0.04	G	5.30×10^{-1}	NS	NS	0.78	0.35	1.74
rs11966079	48383311	6	C6orf138/MUT	T	C	0.14	G	4.78×10^{-1}	NS	NS	0.86	0.56	1.32
rs325286	48387129	6	C6orf138/MUT	T	C	0.45	G	3.34×10^{-2}	NS	NS	0.73	0.54	0.98
rs17762314	64675899	17	PRKCA (within gene)	G	A	0.30	I	4.36×10^{-3}	NS	2.26×10^{-2}	1.57	1.15	2.13
rs11651627	64675914	17	PRKCA (within gene)	T	A	0.38	I	1.04×10^{-1}	NS	NS	0.78	0.58	1.05
rs10491203	64676059	17	PRKCA (within gene)	G	A	0.09	I	1.92×10^{-1}	NS	NS	0.70	0.40	1.22
rs10491202	64676229	17	PRKCA (within gene)	C	G	0.16	I	4.07×10^{-1}	NS	NS	1.18	0.80	1.72
rs8071795	64676380	17	PRKCA (within gene)	C	T	0.09	I	1.02×10^{-1}	NS	NS	0.64	0.37	1.11
rs2052193	64677193	17	PRKCA (within gene)	T	A	0.46	I	8.71×10^{-3}	NS	4.13×10^{-2}	0.68	0.50	0.91
rs9902356	64679742	17	PRKCA (within gene)	C	G	0.12	I	3.47×10^{-4}	3.82×10^{-2}	9.46×10^{-3}	2.12	1.42	3.17
rs11868882	64680301	17	PRKCA (within gene)	G	C	0.22	I	9.76×10^{-1}	NS	NS	0.99	0.70	1.41
rs16960009	64681102	17	PRKCA (within gene)	T	G	0.08	G	4.33×10^{-2}	NS	NS	1.70	1.03	2.81
rs8070293	64682078	17	PRKCA (within gene)	T	C	0.24	I	1.21×10^{-1}	NS	NS	1.30	0.94	1.81
rs8070556	64682079	17	PRKCA (within gene)	T	G	0.06	I	4.68×10^{-1}	NS	NS	0.80	0.43	1.48
rs7224351	64684037	17	PRKCA (within gene)	G	A	0.40	I	1.64×10^{-2}	NS	NS	0.69	0.51	0.94
rs17689224	64684265	17	PRKCA (within gene)	T	C	0.22	I	7.08×10^{-1}	NS	NS	1.07	0.76	1.51
rs1010546	64684565	17	PRKCA (within gene)	T	C	0.14	G	3.29×10^{-1}	NS	NS	1.23	0.82	1.85
rs1010545	64684642	17	PRKCA (within gene)	C	T	0.36	G	3.41×10^{-1}	NS	NS	1.16	0.86	1.56
rs16960016	64685001	17	PRKCA (within gene)	T	C	0.06	I	3.37×10^{-1}	NS	NS	0.74	0.39	1.39

rs2227857	64685078	17	PRKCA (within gene)	A	G	0.36	G	3.01×10^{-1}	NS	NS	1.17	0.87	1.58
rs2286958	64685390	17	PRKCA (within gene)	G	C	0.40	I	1.64×10^{-2}	NS	NS	0.69	0.51	0.94
rs8072511	64686036	17	PRKCA (within gene)	G	C	0.06	I	3.37×10^{-1}	NS	NS	0.74	0.39	1.39
rs8072920	64686048	17	PRKCA (within gene)	G	A	0.40	I	1.64×10^{-2}	NS	NS	0.69	0.51	0.94
rs11869446	64686545	17	PRKCA (within gene)	T	C	0.17	I	6.16×10^{-1}	NS	NS	1.10	0.76	1.61
rs7220480	64686679	17	PRKCA (within gene)	G	A	0.38	I	3.42×10^{-2}	NS	NS	1.37	1.02	1.84
rs78518692	64808034	17	PRKCA	A	G	0.06	G	3.27×10^{-4}	3.60×10^{-2}	1.19×10^{-2}	0.24	0.10	0.61
rs7208993	64809487	17	PRKCA	T	C	0.07	I	2.75×10^{-2}	NS	NS	1.83	1.08	3.10
rs4791033	64810389	17	PRKCA	A	G	0.06	I	1.46×10^{-4}	1.61×10^{-2}	7.96×10^{-3}	0.20	0.07	0.56
rs71379997	64811585	17	PRKCA	G	A	0.07	I	4.28×10^{-2}	NS	NS	1.74	1.03	2.93
rs12603061	64812198	17	PRKCA	A	G	0.42	G	4.07×10^{-2}	NS	NS	1.35	1.01	1.81
rs9910304	64813153	17	PRKCA	A	G	0.07	I	2.72×10^{-2}	NS	NS	1.81	1.08	3.04
rs35200121	64814251	17	PRKCA	A	G	0.07	G	4.28×10^{-2}	NS	NS	1.74	1.03	2.93
rs118009757	64814470	17	PRKCA	A	G	0.05	I	4.33×10^{-4}	4.77×10^{-2}	6.75×10^{-3}	0.22	0.08	0.62
rs36011047	64814481	17	PRKCA	T	C	0.07	I	4.28×10^{-2}	NS	NS	1.74	1.03	2.93
rs9898120	64815009	17	PRKCA	A	G	0.07	I	2.72×10^{-2}	NS	NS	1.81	1.08	3.04
rs28450079	64815228	17	PRKCA	T	C	0.05	I	4.33×10^{-4}	4.77×10^{-2}	6.75×10^{-3}	0.22	0.08	0.62
rs2362711	64815699	17	PRKCA	T	G	0.05	I	4.33×10^{-4}	4.77×10^{-2}	6.75×10^{-3}	0.22	0.08	0.62
rs16960252	64816194	17	PRKCA	A	G	0.07	I	3.45×10^{-2}	NS	NS	1.78	1.06	3.01
rs34169044	64816852	17	PRKCA	A	C	0.07	G	4.08×10^{-2}	NS	NS	1.75	1.04	2.94
rs8077180	64817092	17	PRKCA	C	T	0.07	I	2.72×10^{-2}	NS	NS	1.81	1.08	3.04
rs9892428	64817409	17	PRKCA	A	G	0.07	I	2.72×10^{-2}	NS	NS	1.81	1.08	3.04
rs2086621	84566349	12	SLC6A15	A	G	0.23	I	2.05×10^{-1}	NS	NS	0.80	0.56	1.14
rs2403182	84566386	12	SLC6A15	A	T	0.23	I	2.22×10^{-1}	NS	NS	0.80	0.56	1.15
rs732438	84566672	12	SLC6A15	T	C	0.16	I	1.61×10^{-2}	NS	NS	0.60	0.39	0.92
rs2403183	84567611	12	SLC6A15	G	C	0.50	I	2.24×10^{-3}	NS	1.22×10^{-2}	0.64	0.48	0.85
rs2403184	84567642	12	SLC6A15	G	A	0.50	I	2.24×10^{-3}	NS	1.22×10^{-2}	0.64	0.48	0.85
rs2086622	84567897	12	SLC6A15	A	G	0.23	I	2.22×10^{-1}	NS	NS	0.80	0.56	1.15
rs1384320	84568175	12	SLC6A15	G	C	0.50	I	2.24×10^{-3}	NS	1.22×10^{-2}	0.64	0.48	0.85
rs1384321	84568301	12	SLC6A15	A	C	0.50	I	2.24×10^{-3}	NS	1.22×10^{-2}	0.64	0.48	0.85
rs79588760	84568408	12	SLC6A15	T	C	0.02	I	4.89×10^{-1}	NS	NS	1.49	0.49	4.48
rs1482441	84569019	12	SLC6A15	T	C	0.50	I	2.24×10^{-3}	NS	1.22×10^{-2}	0.64	0.48	0.85
rs7301137	84569037	12	SLC6A15	C	T	0.16	I	1.61×10^{-2}	NS	NS	0.60	0.39	0.92
rs10862831	84570126	12	SLC6A15	T	A	0.50	I	2.24×10^{-3}	NS	1.22×10^{-2}	0.64	0.48	0.85
rs7970932	84570410	12	SLC6A15	T	G	0.33	I	8.12×10^{-2}	NS	NS	0.76	0.55	1.04
rs11116241	84570986	12	SLC6A15	T	G	0.10	I	3.32×10^{-1}	NS	NS	0.78	0.47	1.30
rs77813835	84571186	12	SLC6A15	G	A	0.06	I	2.30×10^{-1}	NS	NS	0.68	0.35	1.30
rs10746314	84571480	12	SLC6A15	A	G	0.23	I	2.22×10^{-1}	NS	NS	0.80	0.56	1.15
rs10735470	84571554	12	SLC6A15	C	T	0.23	I	2.05×10^{-1}	NS	NS	0.80	0.56	1.14
rs10779081	84572013	12	SLC6A15	T	C	0.50	I	2.24×10^{-3}	NS	1.22×10^{-2}	0.64	0.48	0.85
rs7315066	84572979	12	SLC6A15	A	G	0.23	I	2.05×10^{-1}	NS	NS	0.80	0.56	1.14
rs10779082	84573084	12	SLC6A15	G	A	0.17	G	8.43×10^{-2}	NS	NS	1.40	0.96	2.04
rs7315418	84573238	12	SLC6A15	A	G	0.12	I	7.04×10^{-1}	NS	NS	1.09	0.70	1.69

rs10779083	84573381	12	SLC6A15	T	C	0.50	I	2.24×10^{-3}	NS	1.22×10^{-2}	0.64	0.48	0.85
rs79558964	84573419	12	SLC6A15	C	T	0.04	I	1.15×10^{-1}	NS	NS	0.54	0.23	1.22
rs11116242	84574870	12	SLC6A15	A	G	0.10	I	3.32×10^{-1}	NS	NS	0.78	0.47	1.30
rs1482429	84574961	12	SLC6A15	T	C	0.50	I	2.24×10^{-3}	NS	1.22×10^{-2}	0.64	0.48	0.85
rs11058065	125683903	12	AACS	A	G	0.32	G	1.37×10^{-1}	NS	NS	1.26	0.93	1.71
rs35050183	125685296	12	AACS	C	T	0.38	G	4.92×10^{-2}	NS	NS	1.34	1.00	1.80
rs73233312	125685471	12	AACS	T	C	0.06	G	8.86×10^{-4}	NS	1.07×10^{-2}	0.26	0.10	0.67
rs4448747	125696722	12	AACS	A	G	0.28	G	2.37×10^{-1}	NS	NS	1.21	0.88	1.67
rs1571888	125699029	12	AACS	T	C	0.06	G	8.63×10^{-1}	NS	NS	1.05	0.59	1.88
rs4765231	125704267	12	AACS	T	G	0.16	G	4.06×10^{-1}	NS	NS	1.18	0.80	1.73
rs7970411	125706376	12	AACS	G	T	0.06	G	4.82×10^{-1}	NS	NS	1.25	0.68	2.30
rs4442602	125710951	12	AACS	T	C	0.29	G	1.32×10^{-3}	NS	1.44×10^{-2}	1.66	1.22	2.25
rs80131194	125711302	12	AACS	C	T	0.11	G	1.19×10^{-1}	NS	NS	0.68	0.42	1.12
rs4765028	125712728	12	AACS	G	T	0.19	G	2.69×10^{-1}	NS	NS	1.23	0.86	1.76
rs10846850	125715717	12	AACS	T	C	0.13	G	7.65×10^{-4}	NS	1.04×10^{-2}	0.43	0.26	0.73
rs7133139	125719088	12	AACS	A	G	0.23	G	1.41×10^{-1}	NS	NS	1.29	0.92	1.80
rs11058097	125721061	12	TMEM132B/AACS	T	C	0.18	G	1.87×10^{-1}	NS	NS	1.28	0.89	1.85
rs75393874	125724864	12	TMEM132B/AACS	C	T	0.03	G	7.97×10^{-1}	NS	NS	0.89	0.37	2.13
rs12818316	125737328	12	TMEM132B/AACS	T	C	0.27	G	1.00×10^{-2}	NS	4.54×10^{-2}	1.52	1.11	2.08
rs61943077	125746191	12	TMEM132B/AACS	A	G	0.36	G	1.10×10^{-2}	NS	4.79×10^{-2}	0.67	0.49	0.92
rs4765236	125746249	12	TMEM132B/AACS	C	T	0.05	G	1.20×10^{-1}	NS	NS	1.65	0.89	3.08
