# Supplementary material for: Fine-scale haplotype mapping of MUT, AACS, SLC6A15 and PRKCA genes indicates association with insulin resistance of metabolic syndrome and relationship with branched chain amino acid metabolism or regulation
Source: PLoS One. 2019 Mar 26;14(3):e0214122. doi: 10.1371/journal.pone.0214122 (PMC6435171; doi:10.1371/journal.pone.0214122)
Supplement: S6 Table — Genomic regions are indicated in the text and the LD blocking structure is depicted in Fig 2. Only frequent (> 0.1) and significant associated haplotypes are indicated in Table 2. (PDF) [file pone.0214122.s007.pdf]

MUT Block	HaplotypeID	Frequency	P-value (χ^2)
**Block 2**			
CAAAAACGC	B2_H1	0.284	8.80×10^{-1}
CAAAAACGT	B2_H2	0.002	6.12×10^{-1}
CAAAAGCGC	B2_H3	0.001	1.87×10^{-1}
CCAAAACGC	B2_H4	0.046	1.10×10^{-1}
CCAAAGCGC	B2_H5	0.023	4.79×10^{-1}
CCAAAGTGC	B2_H6	0.077	8.97×10^{-2}
CCAAGACGC	B2_H7	0.043	6.66×10^{-2}
CCAAGACGT	B2_H8	0.089	5.86×10^{-2}
CCAAGGTGC	B2_H9	0.001	5.72×10^{-1}
TCGGGACGC	B2_H10	0.001	5.50×10^{-1}
CCGAGACGC	B2_H11	0.052	1.63×10^{-1}
CCGGGACGC	B2_H12	0.128	6.60×10^{-3}
TCGAGACGC	B2_H13	0.216	1.50×10^{-1}
TCGAGACGT	B2_H14	0.002	4.75×10^{-1}
TCGAGACAC	B2_H15	0.028	9.64×10^{-1}
TCGAGGCGC	B2_H16	0.005	9.68×10^{-1}
**Block 3**			
CACTTCAC	B3_H1	0.058	4.42×10^{-1}
CACTTCGC	B3_H2	0.230	1.98×10^{-1}
CATGTCAC	B3_H3	0.214	1.00×10^{-4}
CGCGTTGT	B3_H4	0.002	4.84×10^{-1}
CGCGTCAC	B3_H5	0.037	4.12×10^{-1}
CGCGTTAC	B3_H6	0.190	8.94×10^{-1}
CGCGTTAT	B3_H7	0.077	6.84×10^{-1}
CGCGTTGC	B3_H8	0.002	4.66×10^{-1}
CGCGCCAC	B3_H9	0.110	4.15×10^{-2}
CGCGCTAC	B3_H10	0.001	5.60×10^{-1}
CGTGTTAC	B3_H11	0.001	1.03×10^{-1}
AGCGTCAC	B3_H12	0.076	2.02×10^{-1}
CGCGCCGC	B3_H13	0.001	5.60×10^{-1}
**Block 4**			
ACGACCC	B4_H1	0.360	2.68×10^{-2}
ACGACCT	B4_H2	0.064	3.26×10^{-1}
ACGACTC	B4_H3	0.138	5.17×10^{-1}
ACGGCCC	B4_H4	0.055	2.86×10^{-1}
ATGACCT	B4_H5	0.003	5.05×10^{-1}
ATGATCT	B4_H6	0.033	5.77×10^{-1}
CCGGTCT	B4_H7	0.002	3.86×10^{-1}
CCGACCT	B4_H8	0.217	1.40×10^{-3}
CTGACCC	B4_H9	0.001	5.55×10^{-1}
CCGGCCT	B4_H10	0.002	5.70×10^{-1}
CCAACCT	B4_H11	0.017	5.65×10^{-2}
CTGACCT	B4_H12	0.102	1.71×10^{-1}
CTGATTC	B4_H13	0.001	5.40×10^{-1}
CTGATCT	B4_H14	0.005	8.77×10^{-1}

AACS

Block	HaplotypeID	Frequency	P-value (χ^2)
**Block 17**			
GTCA	B17_H1	0.277	2.16×10^{-1}
GTCG	B17_H2	0.284	7.01×10^{-2}
GTTG	B17_H3	0.057	2.60×10^{-3}
GCCG	B17_H4	0.057	2.63×10^{-1}
ACCG	B17_H5	0.325	1.35×10^{-1}
**Block 18**			
CGTCCT	B18_H1	0.108	1.90×10^{-1}
CGTCTG	B18_H2	0.041	7.09×10^{-1}
CGTCTT	B18_H3	0.349	8.00×10^{-4}
CGTTTT	B18_H4	0.272	1.00×10^{-4}
CGGCTT	B18_H5	0.003	7.47×10^{-1}
CTTCTG	B18_H6	0.144	2.10×10^{-1}
CTTTTT	B18_H7	0.015	1.35×10^{-1}
CTGCTT	B18_H8	0.002	5.22×10^{-1}
TGTCCT	B18_H9	0.003	2.76×10^{-1}
TTTCTG	B18_H10	0.003	7.62×10^{-1}
TGTTTT	B18_H11	0.008	4.36×10^{-1}
TGGCTT	B18_H12	0.049	6.28×10^{-1}
TGTCTG	B18_H13	0.001	1.07×10^{-1}
**Block 19**			
CACTCAT	B19_H1	0.002	3.80×10^{-1}
CACTCGC	B19_H2	0.045	2.11×10^{-1}
CACTTGT	B19_H3	0.003	2.97×10^{-1}
CACCCGT	B19_H4	0.007	4.35×10^{-1}
CATTCAT	B19_H5	0.004	9.10×10^{-1}
CATTCGT	B19_H6	0.164	1.15×10^{-1}
CATCCGT	B19_H7	0.005	9.69×10^{-1}
CGCTCAT	B19_H8	0.345	1.52×10^{-2}
CGCTCGT	B19_H9	0.015	1.50×10^{-2}
CGCTCGC	B19_H10	0.003	1.25×10^{-1}
CGCTTGT	B19_H11	0.257	6.80×10^{-3}
CGCCCAT	B19_H12	0.006	9.01×10^{-1}
CGCCCGT	B19_H13	0.007	6.64×10^{-1}
CGCCTGT	B19_H14	0.003	1.55×10^{-1}
CGTTCGT	B19_H15	0.002	3.99×10^{-1}
TGCTCAT	B19_H16	0.004	5.14×10^{-1}
TGCTCGT	B19_H17	0.119	2.50×10^{-3}
TGCTTGT	B19_H18	0.004	6.32×10^{-1}
CGTCCGT	B19_H19	0.002	4.74×10^{-1}
TACTTGT	B19_H20	0.001	5.51×10^{-1}

SLC6A15

Block	HaplotypeID	Frequency	P-value (χ^2)
**Block 1**			
GTCCAGCCC	B1_H1	0.486	4.00×10^{-3}
GTCCAGCCT	B1_H2	0.015	4.77×10^{-1}
GTCGGGGAC	B1_H3	0.105	6.72×10^{-1}
GTTGGGGAC	B1_H4	0.165	1.93×10^{-2}
ATCGGGGAC	B1_H5	0.001	5.40×10^{-1}
AACGGAGAC	B1_H6	0.228	2.26×10^{-1}
**Block 2**			
CTAGGAGTCG	B2_H1	0.440	3.00×10^{-4}
CTAGGGGTCG	B2_H2	0.061	2.74×10^{-1}
TTTGGAGTTG	B2_H3	0.008	7.54×10^{-2}
TTTTGGGCTA	B2_H4	0.001	5.40×10^{-1}
TTTTGAACTA	B2_H5	0.228	2.26×10^{-1}
TTTTTAGTTG	B2_H6	0.098	3.40×10^{-1}
TCTGGAGTTG	B2_H7	0.165	1.93×10^{-2}
**Block 3**			
AGCTGC	B3_H1	0.293	7.80×10^{-3}
AGCCGC	B3_H2	0.044	1.32×10^{-1}
AGTTGT	B3_H3	0.277	2.10×10^{-3}
AGTTAT	B3_H4	0.098	3.40×10^{-1}
AATTGT	B3_H5	0.119	7.02×10^{-1}
GGCTGC	B3_H6	0.164	9.17×10^{-2}
GGTTGT	B3_H7	0.005	8.60×10^{-1}

PRKCA

Block	HaplotypeID	Frequency	P-value (χ^2)
**Block 65**			
AAACTTGCG	B65_H1	0.002	5.36×10^{-1}
AAAGTAGCG	B65_H2	0.011	6.21×10^{-1}
AAAGTAGGG	B65_H3	0.208	7.09×10^{-1}
AAAGTACCT	B65_H4	0.015	8.91×10^{-1}
AAAGTTGCG	B65_H5	0.002	4.09×10^{-1}
AAGGCTGCG	B65_H6	0.083	1.79×10^{-1}
AAGGCTGGG	B65_H7	0.003	8.17×10^{-1}
ATAGCTGCG	B65_H8	0.005	1.69×10^{-1}
ATAGTAGCG	B65_H9	0.002	5.21×10^{-1}
ATAGTAGGG	B65_H10	0.003	1.13×10^{-1}
ATAGTACCG	B65_H11	0.002	4.71×10^{-1}
ATAGTTGCG	B65_H12	0.364	1.05×10^{-1}
ATAGTTGCT	B65_H13	0.002	4.02×10^{-1}
GAACTAGCG	B65_H14	0.158	7.09×10^{-1}
GAACTACCT	B65_H15	0.004	2.99×10^{-2}
GAAGTAGCG	B65_H16	0.028	9.75×10^{-1}
GAAGTAGGG	B65_H17	0.002	4.62×10^{-1}
GAAGTACCG	B65_H18	0.046	3.80×10^{-3}
GAAGTACCT	B65_H19	0.055	5.37×10^{-2}
GAAGTTGCG	B65_H20	0.001	5.47×10^{-1}
GTAGTACCG	B65_H21	0.001	5.41×10^{-1}

**Block 66**			
CGACCCCACCACG	B66_H1	0.002	4.30×10^{-1}
CGACCTCGCCACG	B66_H2	0.004	8.96×10^{-1}
CGACCTCGCCACA	B66_H3	0.001	5.40×10^{-1}
CGACTCCACCACG	B66_H4	0.072	2.46×10^{-2}
CGATCCCACCACA	B66_H5	0.214	8.88×10^{-1}
CTACTCTACGATG	B66_H6	0.002	5.30×10^{-1}
CGGCCTCGGCGCA	B66_H7	0.398	1.57×10^{-2}
CGGCCTCGGCGTA	B66_H8	0.004	6.13×10^{-1}
CTACTCTACGACG	B66_H9	0.062	2.92×10^{-1}
CTATCCCACCACA	B66_H10	0.001	1.02×10^{-1}
TGACCCCACCATG	B66_H11	0.009	8.69×10^{-1}
TGACCTCGCCACG	B66_H12	0.075	4.31×10^{-2}
TGACCTCGCCATG	B66_H13	0.156	6.51×10^{-1}
**Block 92**			
GCGAA	B92_H1	0.416	3.27×10^{-2}
GCGAG	B92_H2	0.453	1.04×10^{-1}
GCGGA	B92_H3	0.002	3.86×10^{-1}
GTGGG	B92_H4	0.068	2.25×10^{-2}
ACGAG	B92_H5	0.003	8.15×10^{-1}
ACAAG	B92_H6	0.058	7.00×10^{-4}
**Block 93**			
AAGCACGGCCA	B93_H1	0.001	1.03×10^{-1}
AAGTACGAACA	B93_H2	0.068	4.66×10^{-2}
AAGTACGGACA	B93_H3	0.001	5.40×10^{-1}
AGGTACGAACA	B93_H4	0.001	1.03×10^{-1}
GGGCGCGGCTG	B93_H5	0.875	7.08×10^{-1}
GGACGTTGCTG	B93_H6	0.054	1.60×10^{-3}
