# Supplementary material for: Fine-scale haplotype mapping of MUT, AACS, SLC6A15 and PRKCA genes indicates association with insulin resistance of metabolic syndrome and relationship with branched chain amino acid metabolism or regulation
Source: PLoS One. 2019 Mar 26;14(3):e0214122. doi: 10.1371/journal.pone.0214122 (PMC6435171; doi:10.1371/journal.pone.0214122)
Supplement: S7 Table — Data are presented as mean ± SEM. Controls and MetS groups were compared using Mann-Whitney test for numerical variable and χ2 for nominal variable. a, NA stands for non applicable, NS stands for non-significant; b, In defining hyperglycemia, hypertension, high triglycerides and low HDL nominal variable, treatment of pre-diagnosed type 2 diabetes, high blood pressure or dyslipidemia were also considered; c, Insulin resistance was considered as function of HOMAIR values; d, Obesity was considered based on Body Mass Index (BMI) > 30 kg/m2; e, Plasma BCAA levels were available only for 92 subjects. (DOCX) [file pone.0214122.s008.docx]

|  | **CTR** | **MetS** | **P value^a^** |
| --- | --- | --- | --- |
| *n* | 284 | 24 | NA |
| Gender (F/M) | 255/29 | 20/4 | NA |
| Age (years) | 37.4 ± 1.35 | 44.25 ± 2.47 | 0.031 |
| BMI (kg/m^2^) | 24.95 ± 0.38 | 42.19 ± 1.66 | < 0.0001 |
| Waist (cm) | 92.37 ± 1.84 | 117.76 ± 3.32 | < 0.0001 |
| Fasting Glucose (mmol/L) | 4.66 ± 0.13 | 6.18 ± 0.58 | < 0.0002 |
| Fasting insulin (μU/mL) | 11.18 ± 1.50 | 16.65 ± 1.87 | < 0.0001 |
| Hyperglycemia (%)^b^ | 9.51 | 58.33 | NA |
| HOMA_IR_ | 1.96 ± 0.12 | 3.82 ± 0.46 | < 0.0001 |
| Insulin resistance (%)^c^ | 7.04 | 75.0 | 0.0001 |
| SBP (mmHg) | 124.15 ± 5.34 | 143.13 ± 7.20 | NS |
| DBP (mmHg) | 72.07 ± 3.52 | 81.57 ± 3.74 | NS |
| Triglycerides (mmol/L) | 1.23 ± 0.13 | 2.26 ± 0.34 | 0.0002 |
| HDL-cholesterol (mmol/L) | 1.16 ± 0.04 | 1.18 ± 0.07 | NS |
| Obesity (%)^d^ | 18.31 | 95.83 | NA |
| Hypertension (%)^b^ | 34.85 | 75.0 | NA |
| High Triglycerides (%)^b^ | 22.88 | 58.30 | NA |
| Low HDL (%)^b^ | 8.80 | 75.0 | NA |
| Total BCAA (μmol/L)^e^ | 410.27 ± 9.9 | 445.71 ± 22.94 | NS |
| Leucine (μmol/L)^e^ | 123.16 ± 3.39 | 137.18 ± 8.1 | 0.018 |
| Valine (μmol/L)^e^ | 226.77 ± 5.42 | 237.72 ± 11.45 | NS |
| Isoleucine (μmol/L)^e^ | 64.95 ± 1.91 | 70.80 ± 4.32 | NS |
