# Supplementary material for: Fine-scale haplotype mapping of MUT, AACS, SLC6A15 and PRKCA genes indicates association with insulin resistance of metabolic syndrome and relationship with branched chain amino acid metabolism or regulation
Source: PLoS One. 2019 Mar 26;14(3):e0214122. doi: 10.1371/journal.pone.0214122 (PMC6435171; doi:10.1371/journal.pone.0214122)
Supplement: S8 Table — BCAA plasma levels (μmol/L) were evaluated in French population between non-carriers and carriers in ANOVA. Significant values are indicated in bold and statistical significance levels as * for P < 5 x 10^−2^ and ** for P < 5 x 10^−3^. aAssociation of each haplotype with IR (direction) significant (S) or non-significant (NS): +, pathogenic; -, protective; (French) stands for significant only in French population; Independent SNPs with positive association were mapped in corresponding haplotype (underlined and bold) as following: MUT B2_H1 for rs12527508 (A/G), B2_H4 for rs12527508 (A/G), B2_H6 for rs12527508 (A/G), rs62408552 (G/A), rs2503674 (T/C), B2_H12 for rs753849 (G/A), rs17674678 (G/A), B2_H13 for rs1199183 (T/C), rs753849 (G/A), B3_H2 for rs325337 (G/A), AACS B19_H11 for rs12818316 (T/C), SLC6A15 B1_H2 for rs79588760 (T/C), B1_H4 for rs732438 (T/C), B2_H7 for rs7301137 (C/T), B3_H2 for rs79558964 (C/T), PRKCA B65_H6 for rs10491203 (G/A), rs8071795 (C/T), B65_H18 for rs9902356 (C/G), B66_H9 for rs8070556 (T/G), rs1010546 (T/C), rs16960016 (T/C), rs8072511 (G/C). (DOCX) [file pone.0214122.s009.docx]

| **Haplotype ID** | **Sequence** | **Association**  **with IR**  **direction (+/-)^a^** | **Leucine** | | **Valine** | | **Isoleucine** | |
| --- | --- | --- | --- | --- | --- | --- | --- | --- |
|  |  |  | **non-carriers** | **carriers** | **non-carriers** | **carriers** | **non-carriers** | **carriers** |
| ***MUT*** |  |  |  |  |  |  |  |  |
| B2_H1 | CAAA**A**ACGC | + NS | 119.92 ± 3.03 | 128.86 ± 3.93 | 224.66 ± 4.98 | 232.07 ± 4.47 | 63.12 ± 1.70 | **69.19 ± 2.42*** |
| B2_H4 | CCAA**A**ACGC | - NS | 121.64 ± 2.47 | **150.66 ± 9.32*** | 224.93 ± 3.79 | **283.32 ± 2.77*** | 64.31 ± 1.42 | **83.24 ± 5.40*** |
| B2_H6 | CCAA**AGT**GC | - S (French) | 124.16 ± 2.33 | 136.92 ± 6.96 | 226.08 ± 3.58 | **255.71 ± 12.36*** | 65.02 ± 1.27 | **74.98 ± 4.66*** |
| B2_H12 | CC**GG**GACGC | - S | 125.03 ± 2.48 | **105.87 ± 8.15*** | 230.17 ± 3.85 | **204.40 ± 12.20*** | 66.14 ± 1.45 | **56.68 ± 4.49*** |
| B2_H13 | **T**C**G**AGACGC | + NS | 124.94 ± 2.72 | 114.00 ± 5.32 | 229.02 ± 4.02 | 219.06 ± 9.44 | 66.71 ± 1.59 | **58.45 ± 2.77*** |
| B3_H2 | CACTTC**G**C | + NS | 125.12 ± 2.69 | **112.55 ± 5.52*** | 228.61 ± 3.90 | 220.29 ± 10.20 | 66.60 ± 1.59 | **58.31 ± 2.78*** |
| B4_H1 | ACGACCC | + S | 128.00 ± 2.87 | 119.36 ± 3.30 | 230.37 ± 4.31 | 224.20 ± 5.90 | 67.51 ± 1.59 | **62.36 ± 1.84*** |
| ***AACS*** |  |  |  |  |  |  |  |  |
| B17_H1 | GTCA | + S (French) | 127.84 ± 7.77 | 142.52 ± 6.35 | 216.40 ± 13.11 | 250.54 ± 11.10 | 62.63 ± 3.23 | **75.51 ± 2.93*** |
| B18_H3 | CGTCTT | - S | 126.00 ± 2.93 | 117.07 ± 4.24 | 234.15 ± 4.61 | **214.36 ± 6.15*** | 66.42 ± 1.66 | 62.53 ± 2.52 |
| B18_H4 | CGTTTT | + S | 119.18 ± 2.76 | **135.22 ± 4.68*** | 222.36 ± 4.28 | **242.58 ± 7.32*** | 63.78 ± 1.65 | 69.24 ± 2.42 |
| B18_H6 | CTTCTG | + S (French) | 123.80 ± 2.54 | 130.61 ± 4.47 | 225.28 ± 3.90 | 240.60 ± 7.44 | 64.39 ± 1.36 | **71.47 ± 2.76*** |
| B19_H11 | CGCT**T**GT | + S | 119.63 ± 2.72 | **135.02 ± 5.06*** | 223.48 ± 4.22 | 240.11 ± 7.98 | 63.92 ± 1.59 | 69.23 ± 2.91 |
| ***SLC6A15*** |  |  |  |  |  |  |  |  |
| B1_H2 | GTCCAGCC**T** | + NS | 125.62 ± 2.22 | 102.35 ± 20.77 | 229.30 ± 3.47 | **180.02 ± 28.59*** | 65.99 ± 1.24 | 56.35 ± 10.24 |
| B1_H4 | GT**T**GGGGAC | - S | 119.48 ± 2.70 | **136.83 ± 4.96*** | 221.96 ± 4.20 | **248.70 ± 7.21*** | 62.85 ± 1.53 | **74.47 ± 2.97**** |
| B2_H7 | T**C**TGGAGTTG | - S | 119.48 ± 2.70 | **136.83 ± 4.96*** | 221.96 ± 4.20 | **248.70 ± 7.21*** | 62.85 ± 1.53 | **74.47 ± 2.97**** |
| B3_H2 | AGC**C**GC | - NS | 134.17 ± 5.14 | 159.65 ± 24.95 | 230.10 ± 8.84 | **307.85 ± 11.25*** | 68.55 ± 2.44 | 83.75 ± 10.55 |
| B3_H3 | AGTTGT | - S | 120.50 ± 2.94 | 127.35 ± 4.36 | 222.90 ± 4.64 | 235.77 ± 6.22 | 62.99 ± 1.66 | **69.35 ± 2.56*** |
| ***PRKCA*** |  |  |  |  |  |  |  |  |
| B65_H3 | AAAGTAGGG | - NS | 143.70 ± 6.15 | **119.36 ± 7.05*** | 247.72 ± 10.65 | **207.81 ± 13.49*** | 72.01 ± 2.99 | 64.75 ± 3.90 |
| B65_H6 | AA**G**G**C**TGCG | - NS | 124.42 ± 2.48 | **97.26 ± 8.50*** | 229.02 ± 3.81 | **198.34 ± 16.11*** | 66.01 ± 1.44 | **50.08 ± 4.33**** |
| B65_H18 | GAAGTA**C**CG | + S | 124.47 ± 2.26 | 137.84 ± 11.18 | 226.18 ± 3.50 | **265.82 ± 15.99*** | 65.53 ± 1.27 | 71.17 ± 4.79 |
| B66_H9 | C**T**AC**T**C**T**AC**G**ACG | - NS | 124.42 ± 2.47 | **94.14 ± 8.84**** | 229.46 ± 3.80 | **184.28 ± 14.09**** | 66.02 ± 1.43 | **48.14 ± 4.33**** |
| B92_H1 | GCGAA | + S | 127.59 ± 6.14 | **148.16 ± 7.82*** | 220.58 ± 11.23 | **256.16 ± 12.89*** | 67.65 ± 3.27 | 72.74 ± 3.27 |
| B92_H2 | GCGAG | - NS | 147.67 ± 6.70 | **124.78 ± 6.67*** | 251.65 ± 11.38 | 219.00 ± 12.63 | 72.52 ± 3.10 | 67.02 ± 3.54 |
