# Supplementary material for: Fine-scale haplotype mapping of MUT, AACS, SLC6A15 and PRKCA genes indicates association with insulin resistance of metabolic syndrome and relationship with branched chain amino acid metabolism or regulation
Source: PLoS One. 2019 Mar 26;14(3):e0214122. doi: 10.1371/journal.pone.0214122 (PMC6435171; doi:10.1371/journal.pone.0214122)
Supplement: S9 Table — Transcription factor binding motifs changes are indicated for each individual SNPs associated with IR (S5 Table) and/or individual BCAA plasma level (Correlation Trend Test). SNPs are indicated in corresponding haplotypes effective on IR and/or BCAA as shown in Tables 2–4. (DOCX) [file pone.0214122.s010.docx]

| **SNP ID** | **Closest gene** | **Associated**  **parameter** | **Effective**  **haplotype** | **Sequence** | **Regulatory motifs altered** |
| --- | --- | --- | --- | --- | --- |
| rs1199183 | C6orf138/MUT | BCAA | B2_H13 | **T**CGAGACGC | Hsf, RP58 |
| rs753849 | C6orf138/MUT | BCAA | B2_H12  B2_H13 | CC**G**GGACGC  TC**G**AGACGC | None |
| rs12527508 | C6orf138/MUT | BCAA | B2_H1  B2_H4  B2_H6 | CAAA**A**ACGC  CCAA**A**ACGC  CCAA**A**GTGC | HDAC2 |
| rs62408552 | C6orf138/MUT | BCAA | B2_H6 | CCAAA**G**TGC | Hoxa5, Sox, TCF4 |
| rs689261 | C6orf138/MUT | BCAA |  |  | Irf, p300 |
| rs325337 | C6orf138/MUT | BCAA | B3_H2 | CACTTC**G**C | None |
| rs6941242 | C6orf138/MUT | BCAA |  |  | Evi-1 |
| rs10491203 | PRKCA (within gene) | BCAA | B65_H6 | AA**G**GCTGCG | AFP1, Irx, Ncx, Pou2f2, Pou3f4, Sp100 |
| rs8071795 | PRKCA (within gene) | BCAA | B65_H6 | AAGG**C**TGCG | None |
| rs8070556 | PRKCA (within gene) | BCAA | B66_H9 | C**T**ACTCTACGACG | NRSF |
| rs1010546 | PRKCA (within gene) | BCAA | B66_H9 | CTAC**T**CTACGACG | SP2 |
| rs16960016 | PRKCA (within gene) | BCAA | B66_H9 | CTACTC**T**ACGACG | None |
| rs8072511 | PRKCA (within gene) | BCAA | B66_H9 | CTACTCTAC**G**ACG | E2F, HMG-IY, NF-kappaB, PTF1-beta |
| rs79588760 | SLC6A15 | BCAA | B1_H2 | GTCCAGCC**T** | Osr |
| rs7970932 | SLC6A15 | BCAA |  |  | CTCF, Cdc5, DMRT4 |
| rs79558964 | SLC6A15 | BCAA | B3_H2 | AGC**C**GC | Brachyury, E4BP4, Evi-1 |
| rs35050183 | AACS | IR |  |  | Ik-1, Myb |
| rs73233312 | AACS | IR | B17_H3 | GT**T**G | STAT |
| rs4442602 | AACS | IR | B18_H4 | CGT**T**TT | None |
| rs10846850 | AACS | IR | B19_H17 | **T**GCTCGT | MIF-1, RFX5 |
| rs12208936 | C6orf138/MUT | IR |  |  | CEBPA, Cdx2, Hoxb13, Hoxb9, SP1, STAT,Mef2, PRDM1, RXRA, STAT |
| rs2167284 | C6orf138/MUT | IR | B3_H3 | CA**T**GTCAC | BCL, Evi-1, Irf |
| rs325041 | C6orf138/MUT | IR | B4_H8 | **C**CGACCT | Ets, Rad21 |
| rs35163082 | C6orf138/MUT | IR |  |  | Fox, RREB-1 |
| rs325286 | C6orf138/MUT | IR | B4_H8 | CCGACC**T** | ATF4 |
| rs17762314 | PRKCA (within gene) | IR | B65_H18 | **G**AAGTACCG | BATF, CEBPB, Pou5f1, Sox |
| rs2052193 | PRKCA (within gene) | IR |  |  | Mef2, Pax-4, SIX5 |
| rs16960009 | PRKCA (within gene) | IR |  |  | None |
| rs7224351 | PRKCA (within gene) | IR | B66_H7 | CG**G**CCTCGGCGCA | GR, Mrg |
| rs2286958 | PRKCA (within gene) | IR | B66_H7 | CGGCCTCG**G**CGCA | GR, STAT |
| rs8072920 | PRKCA (within gene) | IR | B66_H7 | CGGCCTCGGC**G**CA | Arnt, Gm397, ZEB1 |
| rs7220480 | PRKCA (within gene) | IR | B66_H4  B66_H12 | CGACTCCACCAC**G**  TGACCTCGCCAC**G** | ERalpha-a, Zbtb3, p300 |
| rs78518692 | PRKCA | IR | B92_H6 | **A**CAAG | BCL, Irf, PU.1, Pax-5, SETDB1, STAT |
| rs7208993 | PRKCA | IR | B92_H4 | G**T**GGG | None |
| rs4791033 | PRKCA | IR | B92_H6 | AC**A**AG | Myc, Nrf1 |
| rs71379997 | PRKCA | IR | B92_H4 | GTG**G**G | GATA, TAL1 |
| rs12603061 | PRKCA | IR | B92_H1 | GCGA**A** | Gbx1, Gbx2, Hoxa3, Irf, Isx, Lhx4, Msx-1, SRF |
| rs9910304 | PRKCA | IR | B93_H2 | **A**AGTACGAACA | TCF12, ZEB1 |
| rs35200121 | PRKCA | IR | B93_H2 | A**A**GTACGAACA | HNF1, Hmbox1, Hmx, Nkx3 |
| rs118009757 | PRKCA | IR | B93_H6 | GG**A**CGTTGCTG | Mtf1, RP58, TCF12, ZEB1 |
| rs36011047 | PRKCA | IR | B93_H2 | AAG**T**ACGAACA | None |
| rs9898120 | PRKCA | IR | B93_H2 | AAGT**A**CGAACA | Ets, Lmo2-complex, Mxi1, Myf, Pitx2, ZEB1, Znf143 |
| rs28450079 | PRKCA | IR | B93_H6 | GGACG**T**TGCTG | Zfp410 |
| rs2362711 | PRKCA | IR | B93_H6 | GGACGT**T**GCTG | PU.1 |
| rs16960252 | PRKCA | IR | B93_H2 | AAGTACG**A**ACA | None |
| rs34169044 | PRKCA | IR | B93_H2 | AAGTACGA**A**CA | BCL, NRSF, Sin3Ak-20 |
| rs8077180 | PRKCA | IR | B93_H2 | AAGTACGAA**C**A | HDAC2, Nanog, Pax-5, Pou2f2 |
| rs9892428 | PRKCA | IR | B93_H2 | AAGTACGAAC**A** | None |
| rs2403183 | SLC6A15 | IR | B1_H4 | GTT**G**GGGAC | None |
| rs2403184 | SLC6A15 | IR | B1_H4 | GTTG**G**GGAC | AP-2, EBF, ELF1, Egr-1, YY1 |
| rs1384320 | SLC6A15 | IR | B1_H4 | GTTGGG**G**AC | CTCF, Rad21, SETDB1, SMC3 |
| rs1384321 | SLC6A15 | IR | B1_H4 | GTTGGGG**A**C | BCL, Cdx, Evi-1, GATA, HDAC2, HMGN3, Mef2, Spz1 |
| rs1482441 | SLC6A15 | IR | B2_H7 | **T**CTGGAGTTG | Ets, GATA, Pou6f1, TATA |
| rs10862831 | SLC6A15 | IR | B2_H7 | TC**T**GGAGTTG | Ets |
| rs10779081 | SLC6A15 | IR | B2_H7 | TCTGGAGT**T**G | Hoxa5, RORalpha1, TCF4 |
| rs10779083 | SLC6A15 | IR | B3_H3 | AG**T**TGT | Foxd1, Foxk1, Foxo, Foxq1, HNF1 |
| rs1482429 | SLC6A15 | IR | B3_H3 | AGTTG**T** | Cdx2, Dbx1, Evi-1, Foxa, Foxi1, Foxj2, Foxk1, Foxl1, Foxp1, HNF1, Hoxa10, Hoxa9, Hoxb8,  Hoxb9, Hoxc6, Hoxc9, Hoxd8, Mef2, Ncx, Nkx3, Nkx6-2, Pou2f2, Pou3f3, Pou3f4, Pou6f1, Sox, TATA, Zfp105 |
| rs61943077 | TMEM132B/AACS | IR | B19_H8 | CGCTC**A**T | HNF1, Hoxb13, Hoxd10, Mef2 |
| rs17674678 | C6orf138/MUT | IR+BCAA | B2_H12 | CCG**G**GACGC | CEBPA, CEBPB, p300 |
| rs2503674 | C6orf138/MUT | IR+BCAA | B2_H6 | CCAAAG**T**GC | None |
| rs9902356 | PRKCA (within gene) | IR+BCAA | B65_H18 | GAAGTA**C**CG | TCF11::MafG |
| rs732438 | SLC6A15 | IR+BCAA | B1_H4 | GT**T**GGGGAC | AP-1 |
| rs7301137 | SLC6A15 | IR+BCAA | B2_H7 | T**C**TGGAGTTG | Hmx, Hoxd10, Nkx2, Sox |
| rs12818316 | TMEM132B/AACS | IR+BCAA | B19_H11 | CGCT**T**GT | GR, Pax-5, Znf143 |
